# Sequencing and Comparative Genomic Analysis of a Highly Metal-Tolerant *Penicillium janthinellum* P1 Provide Insights Into Its Metal Tolerance

**DOI:** 10.3389/fmicb.2021.663217

**Published:** 2021-06-04

**Authors:** Bin-Bin Chi, Ya-Nan Lu, Ping-Chuan Yin, Hong-Yan Liu, Hui-Ying Chen, Yang Shan

**Affiliations:** ^1^College of Chemistry and Bioengineering, Guilin University of Technology, Guilin, China; ^2^Guangxi Key Laboratory of Electrochemical and Magneto-Chemical Functional Materials, College of Chemistry and Bioengineering, Guilin University of Technology, Guilin, China; ^3^Hunan Agricultural Product Processing Institute, Hunan Academy of Agricultural Sciences, Changsha, China

**Keywords:** *Penicillium janthinellum*, chromate resistance, whole genome sequence, comparative genomic, gene loci analysis

## Abstract

Heavy metal pollution is a global knotty problem and fungi hold promising potential for the remediation of wastewater containing heavy metals. Here, a new highly chromium-tolerance species, *Penicillium janthinellum* P1, is investigated. The genome of P1 was sequenced and assembled into 30 Mb genome size containing 10,955 predicted protein-coding genes with a GC content of 46.16% through an integrated method of Illumina short-read sequencing and single-molecule real-time Pacific Biosciences sequencing platforms. Through a phylogenetic analysis with model species of fungi, the evolutionary divergence time of *Penicillium janthinellum* P1 and *Penicillium oxalicum* 114-2 was estimated to be 74 MYA. 33 secondary metabolism gene clusters were identified via antiSMASH software, mainly including non-ribosomal peptide synthase genes and T1 polyketide synthase genes. 525 genes were annotated to encode enzymes that act on carbohydrates, involving 101 glucose-degrading enzymes and 24 polysaccharide synthase. By whole-genome sequence analysis, large numbers of metal resistance genes were found in strain P1. Especially ABC transporter and Superoxide dismutase ensure that the P1 fungus can survive in a chromium-polluted environment. ChrA and ChrR were also identified as key genes for chromium resistance. Analysis of their genetic loci revealed that the specific coding-gene arrangement may account for the fungus’s chromium resistance. Genetic information and comparative analysis of *Penicillium janthinellum* are valuable for further understanding the mechanism of high resistance to heavy metal chromium, and gene loci analysis provides a new perspective for identifying chromium-resistant strains.

## Introduction

The discharge of wastes from increased industrialization and human activities has resulted in negative impacts on the environment (Ayres, 1992). Currently, heavy metals have become a severe environmental pollutant and fungi hold promising potential for the remediation of sewage containing heavy metals (Leitao, 2009; Xu et al., 2020). Compared to physicochemical treatment methods, bioremediation has unique advantages, including adequate availability of materials, low cost, and no secondary pollution (Wingenfelder et al., 2005). Bioremediation continues to attract significant attention as a workable solution (Yin et al., 2019; Pathak et al., 2021). The previous studies showed that fungi have strong heavy metal tolerance (Liaquat et al., 2020) and can remove heavy metals through mechanisms such as cell wall adsorption, extracellular immobilization, intracellular bioaccumulation, and intracellular reduction, etc. (Xu et al., 2020). Filamentous fungi usually accumulate metal ions into their mycelia and spores through a mechanism involving the cell wall of the fungus. This mechanism also plays an important role in the existence and performance of the fungus, as well as energy absorption and valence conversion (Dusengemungu et al., 2020). However, we have not yet fully revealed the mechanism of how these fungi are resistant to heavy metal. Therefore, a finished and accurate reference genome will lay a solid foundation for understanding the genomic evolution and identifying functional genes for heavy-metal resistance features.

In 1996, the genome of the first eukaryotic *Saccharomyces cerevisiae* was sequenced and genetically annotated (Nies et al., 1990). As progress advances, *Saccharomyces cerevisiae*, a fungal model organism, has certain limitations (Mattiazzi et al., 2012). In 2000, the United States launched the Fungal Genome Initiative which aimed to promote genome-wide sequencing of important fungal representative species in medicine, agriculture, and industry (Zhou J. F. et al., 2008). Until November 2019, approximately 5,200 fungi genomes had been sequenced throughout the world, with a total of 351 fungal genomic sequences deposited in the NCBI database. A large number of genes in fungi related to characteristic properties, including metal resistance, acid resistance, thermo-resistance, and lignocellulose degradation, etc, have been dissected by genome sequencing and annotation. In recent years, there are an increasing number of studies on the sequencing of the whole genome of *Penicillium* species. Eberhard Karls Universität Tübingen reported the draft genome sequence of *Penicillium islandicum* WF-38-12 (Schafhauser et al., 2015). The Institute of Bioengineering of the Russian Academy of Sciences completed the mitochondrial genome sequencing of *Penicillium* ShG4C acid-resistant strains (Mardanov et al., 2016). The Zhengzhou Tobacco Research Institute depicted the draft genome sequence of *Penicillium verruculosum* strain TS63-9 (Hu et al., 2016). Utrecht University drafted the genome sequence of *Penicillium subrubescens* FBCC1632/CBS132785 (Peng et al., 2017). But among those sequenced *Penicillium* strains, none features a high resistance capability to heavy metals.

ChrA and ChrR genes are well known as the anti-chromium-related genes. ChrA gene is the most widely studied gene encoding Cr efflux protein. In previous studies, researchers believed that ChrA protein, as a membrane protein, could pump out intracellular Cr through proton propulsion to improve Cr tolerance of strains (Nakatsu et al., 2013; Gu et al., 2020). ChrR reduces quinones by simultaneous two-electron transfer, avoiding the formation of highly reactive semiquinone intermediates and producing quinols that promote tolerance of H_2_O_2_ (Gonzalez et al., 2005). Enhancing the activity of ChrR in a chromate-remediating bacterial strain may not only increase the rate of chromate transformation (Baldiris et al., 2018), but it may also augment the capacity of these cells to withstand the unavoidable production of H_2_O_2_ that accompanies chromate reduction.

Due to the ecological niche, the metabolic pathways and genetic background of marine microorganisms are far more fierce and versatile than those of terrestrial microorganisms (Dash et al., 2013; Lopez-Hernandez and Cortes, 2019). Researches on marine microbial genomes may reveal new metabolic mechanisms.

According to our previous studies, the marine-derived *Penicillium janthinellum* P1, isolated from sediment samples by our laboratory, shows the high metal-tolerant ability to a variety of heavy metals [survive at below 400 mg/L Cr (VI)], acid resistance, and strong biosorption capacity (Zhou Y. et al., 2008; Chen et al., 2014, 2019; Lu et al., 2017). To gain more genetic information on *P*. *janthinellum* to determine the critical genes involved in the metal resistance, we here sequenced and assembled the genome of P1, presenting a high-quality genome of the *P*. *janthinellum* P1 using a combined method of sequencing technologies. Then, carbohydrate-active enzyme (CAZyme) genes and secondary metabolism gene clusters were predicted in the assembled genome. Comparative genome analysis of strain P1 with other 24 fungi strains revealed significant genetic variance. Putative genes with respect to chromate resistance such as metal ion transport, metal absorption, and efflux were addressed. The results give a first genetic demonstration of the possible factors that may be responsible for the high levels of chromate resistance in strain P1.

## Materials and Methods

### Genome Sequencing

Whole-genome sequencing was completed by BGI Genomics Co., Ltd. The genomic DNA was extracted from single spore mycelia of the *Penicillium janthinellum* P1. Extracted genomic DNA (Grijseels et al., 2016) then was sequenced using high-throughput Illumina Hiseq 2500 sequencing platform for mate paired-end sequencing together with the PacBio RSII single-molecule real-time sequencing platform, respectively.

### Data Processing

The Illumina raw reads were evaluated using FastQC v0.11.6^1^, and processed by utilizing the NGS QC Toolkit v2.3.3 (Patel and Jain, 2012) with Phred 20 as the cutoff to delete the low-quality reads. The resulting reads with linker and adaptors, with >10% unknown bases (N) were filtered out for further analysis. FastUniq v1.1 (Xu et al., 2012) was employed to remove PCR repeats and then the high quality paired-end reads were obtained for following assembly. An in-house bash5tools.py command of SMRT Analysis was implemented to filter low-quality reads and obtain clean reads of PacBio sequencing.

### Genomic Assembly

The clean reads of the PacBio sequencing were *de novo* assembled to obtain the original assembly contigs by FALCON, then were optimized and upgraded by FinisherSC (Lam et al., 2015) using the clean reads of the PacBio sequencing. Bowtie2 was conducted to map the clean reads of the Illumina sequence back to the PacBio assembly contigs to obtain the sam file. Use Samtools to convert sam files to bam files, and sort and index them. The upgraded assembly contigs and indexed bam file were used as the input file of Pilon to obtain the final assembly. The quality of the assembly was assessed by QUAST (Niegowski and Eshaghi, 2007).

### Gene Prediction

Protein-coding genes in P1 genome were predicted using four softwares including GeneWise^2^ (Madeira et al., 2019), Augustus^3^ (Keller et al., 2011), GeneMark-ES^4^ (Borodovsky and Lomsadze, 2011), and MAKER^5^ (Cantarel et al., 2008). In this paper, Augustus was trained on gene models for P1 by combining the Coprinus gene model and RNA-Seq data of P1. GeneWise and GeneMark-ES with default parameters achieved different gene prediction sets, respectively. Finally, all prediction gene results were integrated by MAKER.

### Gene Function Annotation

The putative proteins of P1 were aligned against the RefSeq non-redundant proteins (NCBI Nr) and the SWISS-PROT Protein Knowledgebase (Swiss-Prot) using BLAST and the HMMER v3.3 (Klingenberg and Marugan-Lobon, 2013) search against the Pfam protein families database (Pfam^6^) (El-Gebali et al., 2019; Mistry et al., 2021) to assign general protein function profiles. The predicted genes were then aligned against the Conserved Domain Database (CDD^7^) using RPS-BLAST. Metabolic pathways of the P1 genome were classified based on the KEGG Ortholog results against the Kyoto Encyclopedia of Genes and Genomes (KEGG^8^). KOG enrichment analysis was assigned based on the best match of alignments against The Clusters of Orthologous Groups of proteins (COG^9^) through BLASTP. RepeatMasker v4.0.7 and RepeatModeler v1.0.5 software were used to filtrate and annotate the retroelements, DNA transposons, simple repeats and low-complexity DNA sequences. Simple sequence repeats (SSRs) were annotated by MISA. The tRNA and anticodons of the P1 genome were predicted by tRNAscan-SE v1.23 (Schattner et al., 2005). rRNA genes were predicted by Barrnap v0.9. Other non-coding RNAs such as sRNA and snRNA were identified using Rfam v9.1. AntiSMASH was used to predict the putative secondary metabolism gene clusters by using genome files and annotation files.

### Phylogenetic Tree Construction and Gene Family Analysis

24 fungal species related to Ascomycota together with P1 were used for phylogenetic analysis (Supplementary Table 7) (Wang et al., 2019). The protein sequences of 25 fungi were compared by BLASTP (e-value < le-5). Then, OrthoMCL under default parameters was used to analyze the blastp results. Batch multiple sequence alignment of these genes were calculated by MAFFT v7.221 software (Katoh and Standley, 2013), subsequently combined into long sequences for each species. The conserved sites of multiple sequence alignment results were extracted using Gblocks v0.91b (parameters -t = p), the best amino acid replacement model was determined by ProTest v3.2. The phylogenetic tree was constructed using RAxML-8.1.24 (bootstraps 1000) by the input of the final alignment sequence. Three pairs of species’ divergence times were estimated by the web^10^ and added three fossil calibration points to the molecular clock analysis (Floudas et al., 2012). The most recent common ancestor (MRCA) of *Aspergillus oryzae* and *Aspergillus terreus* were diverged at 65 MYA. *Gloeophyllum trabeum* and *Saccharomyces cerevisiae* were diverged at 628 MYA, while *Trichoderma parareesei* and *Aspergillus clavatus* were diverged at 343 MYA (Chen et al., 2016). The divergence time of other nodes was calculated by r8s v1.80 (PL method, TN algorithm and the smoothing parameter value set to through cross-validation). The tree was idealized in iTOL^11^. The orthologous gene family expansion was analyzed by CAFE v3.1 based on the ultrametric tree (Han et al., 2013). The OCGs undergone expansion/contraction in P1 were predicted by Swiss-Prot. Genome annotation files and protein sequences of *Penicillium janthinellum* P1, *Penicillium oxalicum* 114-2 and *Penicillium janthinellum* ATCC^12^ were used to perform collinearity analysis via MCScanX software (Wang et al., 2012). Genome comparison was investigated by Mauve v2.3.1^13^.

### CAZyme and Heavy Metal Chromium Resistance Gene Analysis

CAZymes were annotated by dbCAN (covered fraction ratio > 0.3, e-value < 1e-5) and BLASTP against CAZyme database (e-value < 1e-5, maximum hit number set 500). The blastp results are integrated with the HMM results to achieve the final CAZyme annotation. Genes related to heavy metal chromium resistance, which are normally regulated by heavy metal stress, were found and analyzed from the proteins encoded by the P1 genome.

### Gene Locus Analysis

Through swiss-prot annotation of the whole protein group and gene annotation file, the locations of target encoding genes on the scaffold were determined. Analyses of other key genes adjacent to the target genes were conducted to try to interpret the characteristics of the strain. Meanwhile, the target gene’s locus of other strains with similar genes was analyzed to observe the diversity of genes.

## Results

### Statistics

As shown in Table 1, a total of 34,520,396 reads were produced using Illumina sequencing technology, thus yielding 5,178 Mb of raw sequencing data. The results of PacBio Sequel platform were shown in Table 2. The total number of bases measured is about 13 GB.

**TABLE 1:** Statistics of Illumina sequencing data.

**Sample name**	**Insert size (bp)**	**Reads length (bp)**	**Raw data (Mb)**	**Adapter (%)**	**Duplication (%)**	**Total reads**	**Filtered reads (%)**	**Low quality filtered reads (%)**	**Clean data (Mb)**
P1	270	(2 × 150)	5,178	0.83	8.92	34,520,396	11.39	1.62	4,587

**TABLE 2:** Statistics of PacBio Reads sequencing data.

**Sample name**	**Valid ZWM number**	**Subreads number**	**Subreads total bases (bp)**	**Subreads mean length (bp)**	**Subreads N50 (bp)**	**Subreads N90 (bp)**	**Subreads max length (bp)**	**Subreads min length (bp)**
P1	825,497	1,487,295	13,183,008,877	8,863	12,387	4,901	89,375	1,000

### Genome Sequencing and Assembly

The pre-processed Illumina and Pacbio clean data were assembled using SOAPdenovo and FALCON, respectively. The resulting genome size was approximately 31 Mb, resulting in 1,289 contigs with Contig N50 of 50.4 kbp using SOAPdenovo. FALCON pipeline was used for the assembly of PacBio sequencing data, generating 143 contigs with Contig N50 of 3077.5 kbp (Table 3).

**TABLE 3:** The results of the second-generation sequencing, the three- generation sequencing assembly and final Assembly data.

	**Illumina**	**PacBio**	**Final assembly**
Genomic size (Mb)	31	33	30.8
Number of contigs	1289	143	29
Contig N50	50397	3077484	3088621
Contig N75	26995	2473545	2712398
Max contig length	248406	4742194	4750088
GC (%)	46.39	45.25	46.16
N’s per 100 kbp	6.49	0	0
Total length	30998873	33629019	31503521

With third-generation data for assembling and second-generation data for evaluation and correction, the *de novo* genome assembly of *P. janthinellum* P1 with a total length of 30.8 Mb (Table 3) was yielded. A total of 29 contigs were generated with Contig N50 of 3,088.6 kbp (Table 3). This size was consistent with the estimated genome size of 29–34 Mb for three *Penicillium* species (Yang et al., 2016; Peng et al., 2020; Zhang et al., 2020).

### Gene Prediction

10,955 CDS were identified in strain P1 and the average gene length was 1591 bp using GeneWise, Augustus, GeneMark-ES, and MAKER software for gene prediction.

### Genome Annotation

#### Nr Annotation

Through Nr annotation, 3,737 protein-coding genes were annotated, and the top 20 species were selected as candidate species from the annotated species for the construction of the phylogenetic tree (Supplementary Table 1).

#### Pfam Annotation

The Pfam database is a collection of a large number of protein families, each of which is represented by multiple sequence alignments and hidden Markov models (HMM). A total of 6,318 protein-coding genes were annotated using the Pfam database.

#### CDD Annotation

NCBI’s Conservative Domain Database (CDD) is a collection of multiple sequence alignments of ancient domains and full-length proteins, which can provide data on protein retention regions during molecular evolution. Through CDD annotation, a total of 7,618 protein-coding genes were annotated.

#### KEGG Annotation

KEGG is a database resource for understanding the advanced functions and practicality of biological systems such as cells, organisms, and ecosystems from molecular-level information, especially large-scale molecular data sets generated by genome sequencing and other high-throughput. KEGG is a database with three major categories of comprehensive system information, genomic information and chemical information. A total of 1,341 genes were annotated through the KEGG database, involving 293 metabolic pathways (Supplementary Table 2).

#### Swiss-Prot Annotation

A total of 7,877 protein-coding genes were predicted in the Swiss-Prot database, including genes with resistance to chromium. The ChrA was annotated in scaffold 14 with function of reducing chromate accumulation, and essential for chromate resistance. The ChrR in scaffold 16 catalyzed the reduction of quinones, and can also reduce toxic chromate to insoluble and less toxic Cr^3+^ (Cervantes et al., 1990).

#### KOG/COG Annotation

**FIGURE 1:**
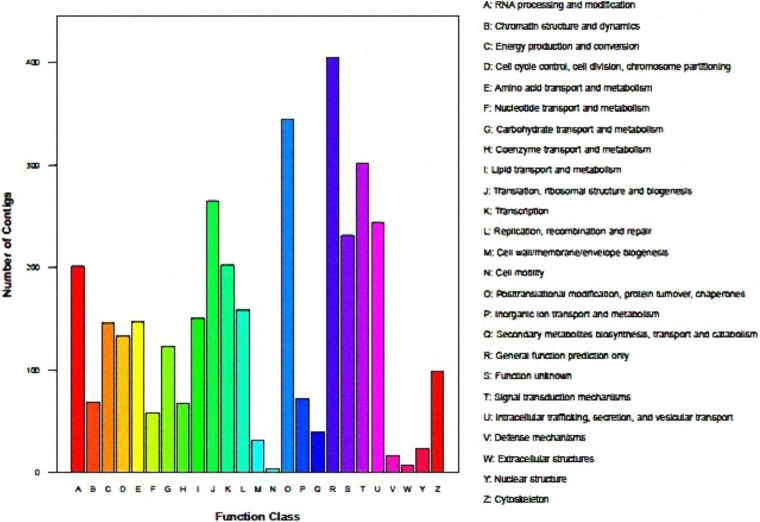
KOG function classification of P1.

Functional clustering diagram is shown as below by analysis of KOG: A (RNA processing and modification), J (translation, ribosomal, structure and biogenesis), K (Transcription), O (Posttranslational, modification, protein turnover, chaperones), T (Signal transduction mechanisms), U (Intracellular trafficking, secretion and vesicular transport). These groups are significant enrichment in genes, and are preferentially enriched in transcriptional secretion and transmembrane protein transport functions. The number of genes in the annotated S (Function unknown) and R (General function prediction only) group is enormous, but the current database cannot clarify their biological functions, indicating that there are still many unknown functions worthy of further elucidating in this regard (Figure 1).

### Repeated Sequence Annotation

Through repeated sequence analysis, there were about 1.90% of the repeated sequences (Supplementary Table 3). Among DNA transposons, Tc1-IS630-Pogo accounts for the largest proportion, amounting to 0.36% of the genome. Among the retrotransposons, LTR transposons are the majority, accounting for 0.18% of the genome.

### Simple Sequence Repeats (SRRs) Annotation

Although simple sequence repeats are usually more likely to occur in non-coding regions, tandem repeats in the coding region of fungal genes cannot be ignored. Research by relevant scholars has shown that SSR occurs in the coding region, which will cause repeated patterns in protein sequences, thus quickly increasing the speed of protein evolution and make fungi adapt to environmental changes faster (Clayton et al., 2017). Therefore, MISA was employed to annotate simple sequence repeats of the coding region of the P1 genome, and the SRR annotation results were shown in Supplementary Table 4.

### Non-coding RNA Annotation

Through analyzing the distribution of tRNA genes, codon usage patterns and amino acid composition at the genomic level is an effective method to decipher the biologically important characteristics of intracellular tRNA and the bias of codon and amino acid usage. A total of 274 tRNAs were annotated, of which there were no false tRNAs (Supplementary Table 5). The number of tRNAs recognizing the same amino acid varied. There were six tRNAs over 20, which were tRNA-Val (27) carrying valine, tRNA-Arg (26) carrying arginine, and glutamic acid. TRNA-Glu (25), tRNA-Ile carrying isoleucine (24), tRNA-Phe carrying phenylalanine (22), tRNA-His carrying glutamic acid (21). The frequency of use of tRNAs carrying anticodons was also more scattered, with 39 groups of tRNAs that recognize the same amino acid carrying the same anticodons.

rRNA is the structural and functional core of the ribosome, which has peptidyl transferase activity. rRNA mainly serves as a binding site for tRNA and a variety of protein synthesis factors, and often binds to mRNA in the extension of peptide chains (Supplementary Table 6).

### Comparative Analysis of *P. janthinellum* P1 With Other Strains

**FIGURE 2:**
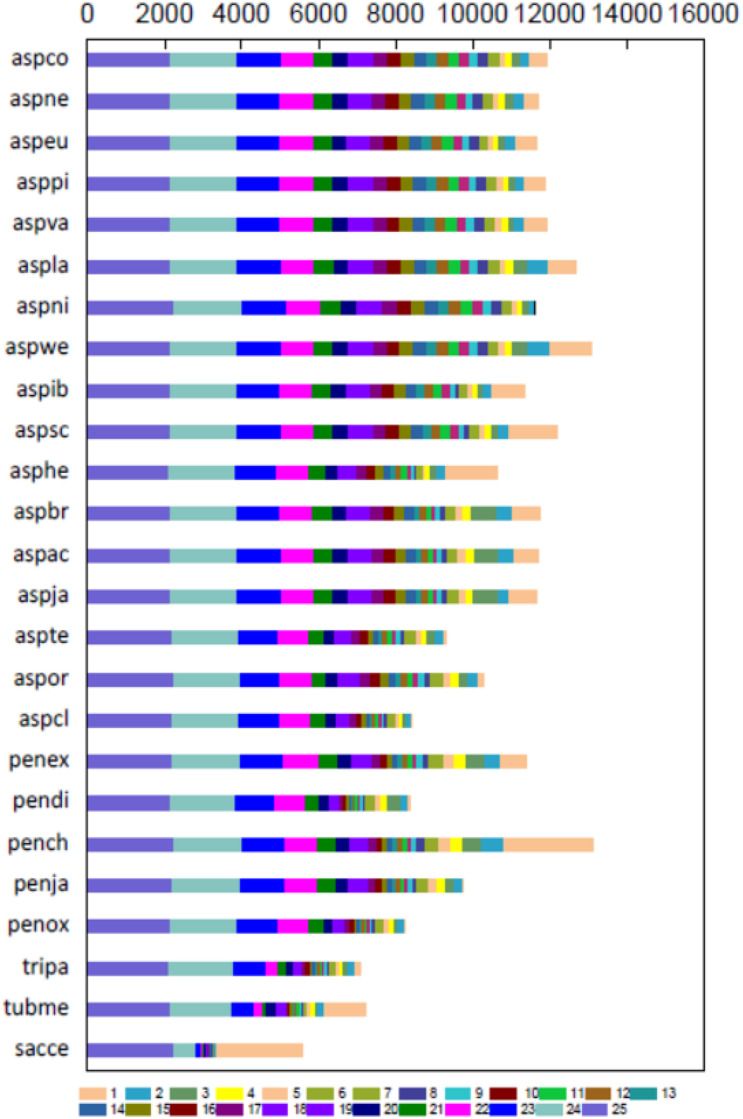
Gene cluster map. Orthologous gene numbers of *Penicillium janthinellum* P1 with other 24 fungal species. The smaller the number of column 1 of a certain species, the higher the degree of similarity with these 25 species.

Total 19150 Ortholog Cluster Groups (OCGs) were constructed, 8764 of which contained 9776 P1 proteins through OrthoMCL analysis. Approximately 20.2% of predicted proteins in P1 had orthotics in all species, whereas 10.8% of proteins were not orthologous to other fungi, and 19.9% of proteins had at least ten paralogs (Figure 2 and Supplementary Table 8), indicating that the P1 strain shares high genetic similarity with the selected *Aspergillus* and *Penicillium* strains.

**FIGURE 3:**
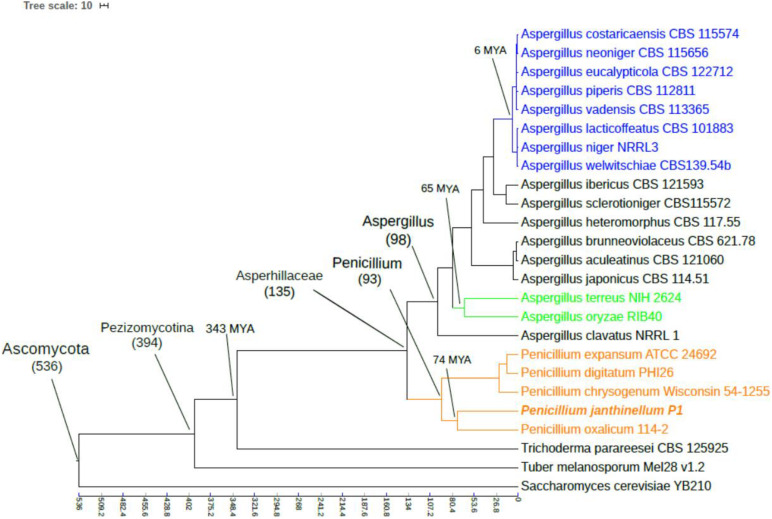
Phylogenetic tree of *Penicillium janthinellum* P1 with other 24 fungal species. The topology of the phylogenetic tree was constructed by the maximum likelihood method (bootstrap = 1000), and all bootstrap values were 100%. Time scale was shown by MYA (million years ago).

The phylogenetic tree was constructed based on 1423 selected single-copy orthologous genes presented in ≥70% of species, revealing the evolutionary history of the P1 strain. The topological structure of the tree was consistent with the taxonomic classification of species (Figure 3). The molecular clock analysis showed that *Penicillium oxalicum* 114-2 had the closest divergence time, which was estimated to be 74 million years ago (MYA). The long divergence time with other strain may be the isolation effect brought by the living environment (Nayfa et al., 2020).

A total of 1183 expansion/contractions family numbers were identified and 102 OCGs were found to have expansion/contraction in P1. After annotation and analysis of those OCGs, it was confirmed that the largest expanded OCG, which encodes Malformin synthetase mlfA, harbors seven genes in P1. The expanded mlfA may indicate that P1 has the potential for biosynthesis of secondary metabolites. Other 17 OCGs including Dynein assembly factor, Aconitate hydratase, Autoinducer 2 sensor kinase/phosphatase, iron transporter, Tol-Pal system protein, oxygen-dependent choline dehydrogenase, high-affinity glucose transporter, L-threonine 3-dehydrogenase, serine/threonine-protein kinase were also expanded in P1. Some enzymes related to respiration have also expanded, which may facilitate strains to resist heavy metal toxicity. Additionally, 306 and 431 syntenic blocks were explored on the basis of the conserved gene order between P1 and *Penicillium oxalicum* 114-2, P1 and *Penicillium janthinellum* ATCC, respectively (Supplementary Figure 1). Average protein similarity between the proteome predicted by P1 with *Penicillium oxalicum* 114-2 and *Penicillium janthinellum* ATCC was evident (Supplementary Figure 2).

### Secondary Metabolism Gene Clusters

A wealth of varied secondary metabolites were found in P1 using the AntiSMASH. A total of 33 secondary metabolism gene clusters were identified. The predicted putative products of 24 secondary metabolism gene clusters were classified into eight different types: seven T1 polyketide synthase (T1pks) gene clusters, seven non-ribosomal peptide synthase (Nrps) gene clusters, two terpene gene clusters, three T1pks-Nrps gene clusters, two fatty acid gene cluster, one Indole-Nrps gene cluster, one saccharide gene cluster, and one Lantipeptide gene cluster; the remaining nine gene clusters synthesized other unknown secondary metabolites (Supplementary Table 12).

### The Secretory System and Transporter

Some microorganisms can secrete extracellular polymeric substances (EPS) such as glycoproteins and soluble amino acids which have the effect of complexing or precipitating metal ions (Naveed et al., 2020). The release of EPS by microorganisms needs the aids of secretory systems and transporters, therefore signal recognition particle (SRP) plays a critical role in the targeting of secretory proteins to cellular membranes (Gowda et al., 1998), and Sec secretion system is responsible for the transport of proteins on the plasma membrane (Fagan and Fairweather, 2011). Within P1 genomic information, 22 proteins were predicted to be components of the eukaryotic Sec-SRP secretion systems (Supplementary Table 9).

### CAZymes

Carbohydrate and energy metabolism acted as key attribution in response to heavy metal adaptation (Kan et al., 2019). Comparing carbohydrate active enzymes of similar fungi may reveal the anti-heavy metal properties of P1. The CAZy database^14^ contains a series of enzymatic catalytic modules and sugar binding modules that degrade, modify or create glycosidic bonds. A total of 525 encoding CAZymes were annotated using dbCAN. The putative CAZymes included 92 Auxiliary Activities (AAs), 44 Carbohydrate-Binding Modules (CBMs), 27 Carbohydrate Esterases (CEs), 250 Glycoside Hydrolases (GHs), 104 GlycosylTransferases (GTs), 8 Polysaccharide Lyases (PLs), of which carbohydrate active enzymes such as glycoside hydrolase were most annotated (Supplementary Table 10). Compared with *P. janthinellum* ATCC 10455 and *P. oxalicum* 114-2, families of AAs in P1 expanded while families of CBMs contracted. AAs acts as redox enzymes that act in conjunction with CAZymes (Supplementary Table 11). This reveals a high oxidoreductase-coding activity in P1, which may be more conducive to the reduction of toxic heavy metals. Cytochrome-c peroxidase of AA2 (EC 1.11.1.5) and p-benzoquinone reductase (NADPH) of AA6 (EC 1.6.5.6) were identified within the P1 genome which were anti-chromium associated proteins. CBM1 as an important cellulose-binding domain family in cellulose degradation was rare in the P1 fungal genome, inferring that it may be related to marine living environment.

Further analyses revealed that 190 CAZymes were predicted to be sugar metabolism enzymes, involving 68 polysaccharide degrading enzymes, 100 glucose degrading enzymes and 12 polysaccharide synthases. The P1 genome was enriched in glucose degrading enzymes assigned to 10 predicted CAZyme families. This finding was compared to 99 genes in *P. janthinellum* ATCC 10455 and 54 genes in *P. oxalicum* 114-2. Compared with the above two strains, larger numbers of genes encoding the important glucose oxidase were classified into AA3 in the P1 genome. This result supports the high sugar metabolism activity of P1, and provides a certain insight into anti-metal activity.

### Heavy Metal Chromium Resistance Gene

The maximum Cr (VI) biosorption capacity for living fungus P1 pellets was about 87% at the optimum condition (Chen et al., 2019), demonstrating strong chromium removal ability (Sriharsha et al., 2020). By analyzing the genes annotated in the P1 genome, the genes that may be related to their high resistance to heavy metal chromium are listed below (Table 4). As we can see, cytochrome P450s (CYPs) were heavily annotated in P1. As a multicomponent electron transport chain system, CYPs were critical in degradation, detoxification, and syntheses of life-critical compounds in organisms (Deng et al., 2007). In addition, CYPs were also found in *Phanerochaete chrysosporium* and the unique P450s had a potential for the production of useful flavonoids (Kasai et al., 2010). Superoxide dismutase can eliminate radicals (Liu et al., 2020), which may be involved in the process of reducing Cr (VI) by fungi.

**TABLE 4:** Putative genes with heavy metal chromium resistance in P1.

**Annotation of heavy metal resistance related genes**	**Predicted number**	**Function**
Cytochrome P450	81	Monooxygenase involved in the metabolism of various endogenous substrates
ABC transporter	71	Transport metabolites and enzyme cofactors (Li et al., 2012), and mediate glutathione-dependent resistance to heavy metals
Superoxide dismutase	42	Destroy free radicals that are normally produced in cells and are toxic to biological systems.
Phate transporter	34	The sulfate transporter absorbs chromate.
WD repeat protein	27	Participate in the processes of transcription regulation, signal transduction, cell proliferation, etc.
Hsp70 chaperone	31	Transcription of heavy metal stress-resistant proteins.
Hsp90 chaperone	12	Related to heavy metal resistance mechanism.
RecF/RecN/SMC protein	9	DNA replication and repair.
Glutathione *S*-transferase	6	Eliminate toxic substances, both internal and external, in living organisms.
C_2_H_2_ transcription factor (Rpn4)	3	Protein degradation
Putative DNA mismatch repair protein Msh2	3	DNA mismatch repair.
Chromate transport	3	Chromate ion transport
FMN-linked oxidoreductase	2	NADH reductase
DNA replication factor C subunit Rfc5	1	DNA replication
Transcription factor AP-1/Yap1	1	Expression of genes with oxidative stress defense and sulfur/glutathione metabolism functions
Copper resistance protein Crd2	1	Related to copper resistance
RAD52 DNA repair protein RADC	1	DNA repair
Citrate synthase Cit1	1	TCA cycle enzyme citrate synthase
Metal homeostasis protein Bsd2	1	Degradation of heavy metal ions (such as cadmium, cobalt and copper)
Ornithine aminotransferase	1	Catalyzes the interconversion of ornithine to glutamate semialdehyde
Chromate transport protein	1	Reduces chromate accumulation
Quinone reductase	1	Catalyzes the reduction of quinones
SWI/SNF family of DNA-dependent ATPase	1	ATP-dependent chromatin remodeling complex

### ChrA and ChrR Gene Loci Analysis

**FIGURE 4:**
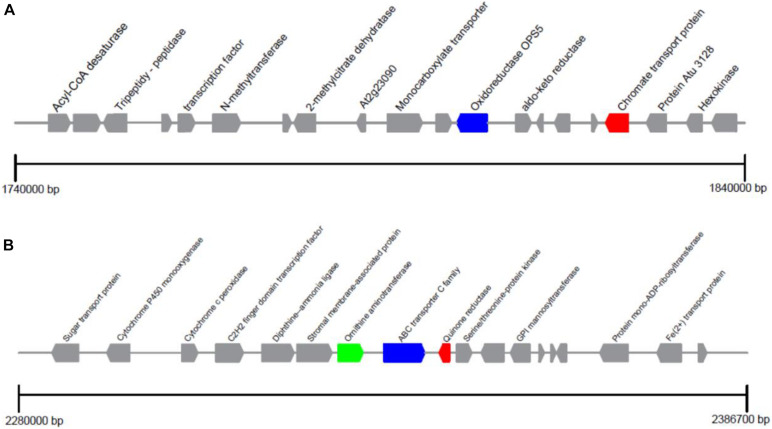
Distribution of genes in the ChrA and ChrB loci of *Penicillium janthinellum* P1. **(A)** ChrA encoding chromate transport protein and **(B)** ChrR encoding quinone reductase loci are positioned on scaffold 14 and 16, respectively.

The encoding genes flanking to the ChrA and ChrR gene loci were studied in detail, seen in Figure 4. ChrA gene located on scaffold 14 can encode chromate transport protein. OpS5 acted as an oxidoreductase is attributed to secondary metabolite biosynthesis. Its physical distance from ChrA gene is about 5.6 Kb. ChrA gene is also present in other similar fungi, and a number of oxidoreductases are present around it. ChrAs in *P. aeruginosa* (Cervantes et al., 1990), *A. eutrophus* (Nies et al., 1990), *Shewanella sp*. (Aguilar-Barajas et al., 2008) have been studied in detail. These studies examined the resistance of direct homologous microorganisms carrying the ChrA gene to different Cr (VI) concentrations, and found that the ChrA gene alone could not explain the different Cr (VI) resistance of different microorganisms.

The ChrR gene is located on scaffold 16 and can encode quinone reductase. Adjacent to the ChrR, the Ornithine aminotransferase RocD and ABC Transporter C family (ABCC) were identified. Interestingly, the same genetic arrangement was found in *Penicillium oxalicum* 114-2, *Penicillium expansum* ATCC 24692, and *Penicillium chrysogenum* Wisconsin 54-1255, and these three genes were arranged in the same order. Part of *Penicillium* species are resistant to chromium (Fukuda et al., 2008; Zhang et al., 2011; Long et al., 2020), which may be explained by the special gene arrangement to a certain extent. In addition, Ornithine aminotransferase catalyzes the conversion of Ornithine into glutamic semialdehyde which subsequently can catalyze the reduction of NADP^+^ to NADPH. The generated NADP^+^ and NADPH will then serve as cofactors of ChrR to catalyze the reduction of Cr (VI). Quinone reductase acted as Cr (VI) reductase was confirmed to be an NADH-dependent reductase and also found in *L. fusiformis* ZC1 (He et al., 2011). ABCC mediates the transport of glutathione (GSH), also be known as an important antioxidant, which can eliminate the ROS produced by the ChrR enzyme during the reduction of Cr ions. GSH is crucial for chromium resistance to microorganisms. Glutathione synthase and glutathione s-transferase were up-regulated under chromium pressure in *P. aeruginosa* (Kılıç et al., 2010). Also the increase of Cr (VI) toxicity in *E. coli* was reported due to the absence of GSH (Helbig et al., 2008). ChrR gene, RocD and ABCC also exist in *Trichoderma parareesei* CBS 125925 and *Saccharomyces cerevisiae* YB210, but they are not on the same scaffold and far apart. We hypothesis this potentially may be a reason for differences in chromium resistance. ABCC alone was not related to the presence of heavy metal-related genes. In summary, it is noteworthy that whether the target resistance gene has such a genetic arrangement may be used as a basis for judging whether the strain is resistant to heavy-metal chromium at the genetic level.

## Discussion

All life phenomena are intimately connected with genetic material. The high quality and accuracy genetic information of filamentous fungi can reveal the mechanism of heavy-metal resistance, and contribute to genetic manipulation of diverse industrial microbial strains for improving the efficiency of bioremediation sewage treatment. Here, we evaluated factors related to chromium resistance at the genomic level. Illumina and PacBio sequencing technologies are now widely used to assemble genomes due to their economy and efficiency (Ma et al., 2021; Yan et al., 2021). In this study, the Contig N50 length of Illumina sequencing technology was relatively short, which was probably related to the high percentage of repetitive sequences, whereas the third-generation sequencing technology can be more efficient in solving the high repeat ratio problem. After comprehensively considering the pros and cons of different sequencing technologies, we adopted an integrated strategy of hybrid assembly that the third-generation sequencing data was mainly utilized to assemble into chromosome sequence, while the second-generation sequencing data for error correction. Thus, the total *de novo* assembled genome size was 30.8 Mb, including 29 contigs with Contig N50 of 3088.6 kbp. By comparing different sets of assembly results, it was found that the hybrid assembly result was significantly better than any sole assembly method. To our knowledge, this is the first report of genome assembly of *P. janthinellum* using combined Illumina and PacBio sequencing platforms.

Genome annotation was implemented through a series of various databases, so that the characteristics of the genome could be precisely evaluated from all different aspects. In the end, a total of 10,955 protein-coding genes within P1 genome was annotated, about 1.90% of which were repetitive sequences, mainly involving simple repetitive sequences, together with 503 tRNA and 274 rRNA.

Metal exposure can lead to a significant inhibition in respiratory enzyme activities as well as corresponding transcript level (Samanta et al., 2020). A large inventory of CAZymes was found, including sugar metabolism enzymes which may ensure respiratory metabolism under heavy metal stress, supporting the high energy metabolism activity of this species. *P. janthinellum* P1 had more T1PKs than *P. oxalicum* sp. And many Sec-SRP secretion proteins were identified in the analysis of the secretory system and transporter. These results suggested that P1 has potential for producing bioactive secondary metabolites.

Our research confirms that P1 possesses the genes encoding proteins required for chromate reduction and efflux, namely ChrR, ChrA, respectively, which could be responsible for chromate resistance in this fungus. Through the analyses of the loci of ChrA and ChrR and the special genes in the vicinity of these loci, together with the annotation of other non-chromium resistant strains, it is found out that chromium resistance of microorganisms may be related to the gene arrangement around the anti-chromium genes. Heavy metal can induce mRNA transcription of Cytochrome P450s (CYPs) enzymes (Sun et al., 2020) and CYPs play diverse roles in metabolism including the synthesis of secondary metabolites as well as the degradation of recalcitrant organic substrates (Linder, 2019). The large amount of CYPs might be essential for the life cycle of *P. janthinellum* P1 and the synthesis of the metabolic products. The microorganism exhibited high oxidative stress supported by a high concentration of reactive oxygen species (ROS) and low levels of reduced glutathione (GSH) in the presence of heavy metals (Caldeira et al., 2021). Biological cells improved the antioxidant capacity by increasing the activities of superoxide dismutase, catalase, and glutathione production (Qi et al., 2021). In addition, Heavy metals can participate in Fenton-like reactions to generate more ROS, which is a direct factor in inducing oxidative stress (Liochev, 1999) and can interact directly with antioxidant enzymes (Gurer-Orhan et al., 2004). In summary, large numbers of identified superoxide dismutases may directly participate in the reduction of heavy metal chromium and reduce toxicity.

## Conclusion

In conclusion, we studied in detail the putative genes in *P. janthinellum* P1 isolated from the ocean that may be involved in important traits of resistance to heavy metals using whole-genome sequencing and comparative genome analysis. The results of this study can facilitate us to further understand the genetic diversity of *P. janthinellum* and shed light on its chromium resistance. The follow-up research work will analyze these in depth from the perspective of transcriptome and proteome level, and further explore mechanism related to heavy metal chromium resistance.

## Data Availability Statement

The datasets presented in this study can be found in online repositories, SRA SRR13016573 and SRR13016574.

## Author Contributions

H-YC and P-CY conceived and designed the experiments. B-BC analyzed the data. H-YC contributed materials and analysis tools. B-BC and Y-NL wrote the manuscript. H-YL and YS revised the manuscript. All authors contributed to the article and approved the submitted version.

## Conflict of Interest

The authors declare that the research was conducted in the absence of any commercial or financial relationships that could be construed as a potential conflict of interest.

## Footnotes

1 https://www.bioinformatics.babraham.ac.uk/projects/fastqc/

2 http://www.ebi.ac.uk/Tools/psa/genewise/

3 http://bioinf.uni-greifswald.de/augustus/

4 http://exon.gatech.edu/GeneMark/

5 http://gmod.org.wiki.MAKER

6 https://pfam.xfam.org

7 http://www.ncbi.nlm.nih.gov/Structure/cdd/wrpsb.cgi

8 http://www.genome.jp/kegg/

9 http://www.ncbi.nlm.nih.gov/COG/

10 http://www.timetree.org/

11 https://itol.embl.de/

12 https://mycocosm.jgi.doe.gov/Penja1/Penja1.home.html

13 http://darlinglab.org/mauve/mauve.html

14 http://www.cazy.org/

## References

[B1] Aguilar-Barajas E. Paluscio E. Cervantes C. Rensing C. (2008). Expression of chromate resistance genes from Shewanella sp. strain ANA-3 in *Escherichia coli*. *FEMS Microbiol. Lett.* 285 97–100. 10.1111/j.1574-6968.2008.01220.x 18537831

[B2] Ayres R. U. (1992). Toxic Heavy Metals: Materials Cycle Optimization. *PNAS* 89 815–820. 10.1073/pnas.89.3.815 11607259 PMC48332

[B3] Baldiris R. Acosta-Tapia N. Montes A. Hernandez J. Vivas-Reyes R. (2018). Reduction of Hexavalent Chromium and Detection of Chromate Reductase (ChrR) in *Stenotrophomonas* maltophilia. *Molecules* 23:23020406. 10.3390/molecules23020406 29438314 PMC6017488

[B4] Borodovsky M. Lomsadze A. (2011). Eukaryotic gene prediction using GeneMark.hmm-E and GeneMark-ES. *Curr. Protoc. Bioinformat.* 6 1–10. 10.1002/0471250953.bi0406s35 21901742 PMC3204378

[B5] Caldeira J. B. Chung A. P. Morais P. V. Branco R. (2021). Relevance of FeoAB system in Rhodanobacter sp. B2A1Ga4 resistance to heavy metals, aluminium, gallium, and indium. *Appl. Microbiol. Biotechnol.* 14 3301–3314. 10.1007/s00253-021-11254-6 33791837

[B6] Cantarel B. L. Korf I. Robb S. M. Parra G. Ross E. (2008). MAKER: An easy-to-use annotation pipeline designed for emerging model organism genomes. *Genome Res.* 18 188–196. 10.1101/gr.6743907 18025269 PMC2134774

[B7] Cervantes C. Ohtake H. Chu L. Misra T. K. Silver S. (1990). Cloning, nucleotide sequence, and expression of the chromate resistance determinant of *Pseudomonas aeruginosa* plasmid pUM505. *J. Bacteriol.* 172 287–291. 10.1128/jb.172.1.287-291.1990 2152903 PMC208430

[B8] Chen H. Y. Guan Y. X. Yao S. J. (2014). A novel two-species whole-cell immobilization system composed of marine-derived fungi and its application in wastewater treatment. *J. Chemical Technol. Biotechnol.* 89 1733–1740. 10.1002/jctb.4253

[B9] Chen H. Y. Lu Y. N. Yin P. C. Li X. Shan Y. (2019). Exploring the Mechanisms of Biosorption of Cr(VI) by Marine-Derived Penicillium janthinellum P1. *Int. J. Agricult. Biol.* 22 913–920. 10.17957/ijab/15.1148

[B10] Chen L. F. Gong Y. H. Cai Y. L. Liu W. Zhou Y. Xiao Y. (2016). Genome Sequence of the Edible Cultivated Mushroom Lentinula edodes (Shiitake) Reveals Insights into Lignocellulose Degradation. *PLoS One* 11:20. 10.1371/journal.pone.0160336 27500531 PMC4976891

[B11] Clayton W. Eaton C. J. Dupont P. Y. Gillanders T. Cameron N. Saikia S. (2017). Analysis of simple sequence repeat (SSR) structure and sequence within Epichloe endophyte genomes reveals impacts on gene structure and insights into ancestral hybridization events. *PLoS One* 12:183748. 10.1371/journal.pone.0183748 28886068 PMC5590859

[B12] Dash H. R. Mangwani N. Chakraborty J. Kumari S. Das S. (2013). Marine bacteria: potential candidates for enhanced bioremediation. *Appl. Microbiol. Biotechnol*. 97 561–571. 10.1007/s00253-012-4584-0 23212672

[B13] Deng J. X. Carbone L. Dean R. A. (2007). The evolutionary history of Cytochrome P450 genes in four filamentous Ascomycetes. *BMC Evol. Biol.* 7:30. 10.1186/1471-2148-7-30 17324274 PMC1828051

[B14] Dusengemungu L. Kasali G. Gwanama C. Ouma K. O. (2020). Recent Advances in Biosorption of Copper and Cobalt by Filamentous Fungi. *Front. Microbiol.* 11:582016. 10.3389/fmicb.2020.582016 33408701 PMC7779407

[B15] El-Gebali S. Mistry J. Bateman A. Eddy S. R. Luciani A. Potter S. C. (2019). The Pfam protein families database in 2019. *Nucleic Acids Res.* 47 D427–D432. 10.1093/nar/gky995 30357350 PMC6324024

[B16] Fagan R. P. Fairweather N. F. (2011). Clostridium difficile Has Two Parallel and Essential Sec Secretion Systems. *J. Biol. Chem.* 286 27483–27493. 10.1074/jbc.M111.263889 21659510 PMC3149341

[B17] Floudas D. Binder M. Riley R. Barry K. Blanchette R. A. Henrissat B. (2012). The Paleozoic Origin of Enzymatic Lignin Decomposition Reconstructed from 31 Fungal Genomes. *Science* 336 1715–1719. 10.1126/science.1221748 22745431

[B18] Fukuda T. Ishino Y. Ogawa A. Tsutsumi K. Morita H. (2008). Cr(VI) reduction from contaminated soils by Aspergillus sp N2 and Penicillium sp N3 isolated from chromium deposits. *J. Gen. Appl. Microbiol.* 54 295–303. 10.2323/jgam.54.295 19029771

[B19] Gonzalez C. F. Ackerley D. F. Lynch S. V. Matin A. (2005). ChrR, a soluble quinone reductase of *Pseudomonas* putida that defends against H2O2. *J. Biol. Chem.* 280 22590–22595. 10.1074/jbc.M501654200 15840577

[B20] Gowda K. Black S. D. Moller I. Sakakibara Y. Liu M. C. Zwieb C. (1998). Protein SRP54 of human signal recognition particle: cloning, expression, and comparative analysis of functional sites. *Gene* 207 197–207. 10.1016/s0378-1119(97)00627-6 9511762

[B21] Grijseels S. Nielsen J. C. Randelovic M. Nielsen J. Nielsen K. F. Workman M. (2016). Penicillium arizonense, a new, genome sequenced fungal species, reveals a high chemical diversity in secreted metabolites. *Sci. Rep.* 6:35112. 10.1038/srep35112 27739446 PMC5064400

[B22] Gu R. J. Gao J. Y. Dong L. L. Liu Y. Li X. L. Bai Q. H. (2020). Chromium metabolism characteristics of coexpression of ChrA and ChrT gene. *Ecotoxicol. Environ. Saf.* 204:111060. 10.1016/j.ecoenv.2020.111060 32768747

[B23] Gurer-Orhan H. Sabir H. U. Ozgunes H. (2004). Correlation between clinical indicators of lead poisoning and oxidative stress parameters in controls and lead-exposed workers. *Toxicology* 195 147–154. 10.1016/j.tox.2003.09.009 14751670

[B24] Han M. V. Thomas G. W. Lugo-Martinez J. Hahn M. W. (2013). Estimating gene gain and loss rates in the presence of error in genome assembly and annotation using CAFE 3. *Mol. Biol. Evol.* 30 1987–1997. 10.1093/molbev/mst100 23709260

[B25] He M. Y. Li X. Y. Liu H. L. Miller S. J. Wang G. J. Rensing C. (2011). Characterization and genomic analysis of a highly chromate resistant and reducing bacterial strain Lysinibacillus fusiformis ZC1. *J. Hazard. Mater.* 185 682–688. 10.1016/j.jhazmat.2010.09.072 20952126

[B26] Helbig K. Bleuel C. Krauss G. J. Nies D. H. (2008). Glutathione and Transition-Metal Homeostasis in *Escherichia coli*. *J. Bacteriol.* 190 5431–5438. 10.1128/jb.00271-08 18539744 PMC2493246

[B27] Hu L. Taujale R. Liu F. Song J. Yin Q. Zhang Y. (2016). Draft genome sequence of Talaromyces verruculosus (”Penicillium verruculosum”) strain TS63-9, a fungus with great potential for industrial production of polysaccharide-degrading enzymes. *J. Biotechnol.* 219 5–6. 10.1016/j.jbiotec.2015.12.017 26707807

[B28] Kan G. F. Wang X. F. Jiang J. Zhang C. S. Chi M. L. Ju Y. (2019). Copper stress response in yeast Rhodotorula mucilaginosa AN 5 isolated from sea ice, Antarctic. *MicrobiologyOpen* 8:657. 10.1002/mbo3.657 29926536 PMC6436437

[B29] Kasai N. Ikushiro S. Hirosue S. Arisawa A. Ichinose H. Uchida Y. (2010). Atypical kinetics of cytochromes P450 catalysing 3’-hydroxylation of flavone from the white-rot fungus Phanerochaete chrysosporium. *J. Biochem.* 147 117–125. 10.1093/jb/mvp155 19819902

[B30] Katoh K. Standley D. M. (2013). MAFFT multiple sequence alignment software version 7: improvements in performance and usability. *Mol. Biol. Evol.* 30 772–780. 10.1093/molbev/mst010 23329690 PMC3603318

[B31] Keller O. Kollmar M. Stanke M. Waack S. (2011). A novel hybrid gene prediction method employing protein multiple sequence alignments. *Bioinformatics* 27 757–763. 10.1093/bioinformatics/btr010 21216780

[B32] Kılıç N. K. Stensballe A. Otzen D. E. Dönmez G. (2010). Proteomic changes in response to chromium(VI) toxicity in *Pseudomonas aeruginosa*. *Bioresour. Technol.* 101 2134–2140. 10.1016/j.biortech.2009.11.008 19945860

[B33] Klingenberg C. P. Marugan-Lobon J. (2013). Evolutionary Covariation in Geometric Morphometric Data: Analyzing Integration, Modularity, and Allometry in a Phylogenetic Context. *Syst. Biol.* 62 591–610. 10.1093/sysbio/syt025 23589497

[B34] Lam K. K. LaButti K. Khalak A. Tse D. (2015). FinisherSC: a repeat-aware tool for upgrading de novo assembly using long reads. *Bioinformatics* 31 3207–3209. 10.1093/bioinformatics/btv280 26040454

[B35] Leitao A. L. (2009). Potential of Penicillium Species in the Bioremediation Field. *Int. J. Env. Res. Public Health* 6 1393–1417. 10.3390/ijerph6041393 19440525 PMC2681198

[B36] Li W. L. Cong Q. Pei J. M. Kinch L. N. Grishin N. V. (2012). The ABC transporters in Candidatus Liberibacter asiaticus. *Proteins Struct. Funct. Bioinformat.* 80 2614–2628. 10.1002/prot.24147 22807026 PMC3688454

[B37] Liaquat F. Munis M. F. H. Haroon U. Arif S. Saqib S. Zaman W. (2020). Evaluation of Metal Tolerance of Fungal Strains Isolated from Contaminated Mining Soil of Nanjing. China. *Biology-Basel* 9:12. 10.3390/biology9120469 33333787 PMC7765179

[B38] Linder T. (2019). Taxonomic Distribution of Cytochrome P450 Monooxygenases (CYPs) among the Budding Yeasts (Sub-Phylum Saccharomycotina). *Microorganisms* 7:20. 10.3390/microorganisms7080247 31398949 PMC6723986

[B39] Liochev S. I. (1999). “The mechanism of “Fenton-like” reactions and their importance for biological systems. A biologist’s view,” in *Metal Ions in Biological Systems, Vol 36: Interrelations between Free Radicals and Metal Ions in Life Processes*, eds Sigel A. Sigel H. (New York, NY: Marcel Dekker), 1–39. 10093922

[B40] Liu L. Y. Cui H. N. Xu Y. (2020). Quantitative Estimation of Oxidative Stress in Cancer Tissue Cells Through Gene Expression Data Analyses. *Front. Genet.* 11:494. 10.3389/fgene.2020.00494 32528526 PMC7263278

[B41] Long B. B. Ye J. E. Ye Z. He J. Y. Luo Y. T. Zhao Y. G. (2020). Cr(VI) removal by Penicillium oxalicum SL2: Reduction with acidic metabolites and form transformation in the mycelium. *Chemosphere* 253:126731. 10.1016/j.chemosphere.2020.126731 32302907

[B42] Lopez-Hernandez F. Cortes A. J. (2019). Last-Generation Genome-Environment Associations Reveal the Genetic Basis of Heat Tolerance in Common Bean (Phaseolus vulgaris L.). *Front. Genet.* 10:954. 10.3389/fgene.2019.00954 31824551 PMC6883007

[B43] Lu T. Zhang Q. L. Yao S. J. (2017). Efficient decolorization of dye-containing wastewater using mycelial pellets formed of marine-derived Aspergillus niger. *Chin. J. Chem. Eng.* 25 330–337. 10.1016/j.cjche.2016.08.010

[B44] Ma D. N. Guo Z. J. Ding Q. S. Zhao Z. Z. Shen Z. J. Wei M. Y. (2021). Chromosome-level assembly of the mangrove plant Aegiceras corniculatum genome generated through Illumina, PacBio and Hi-C sequencing technologies. *Mol. Ecol. Resour.* 15:13347. 10.1111/1755-0998.13347 33550674

[B45] Madeira F. Park Y. M. Lee J. Buso N. Gur T. Madhusoodanan N. (2019). The EMBL-EBI search and sequence analysis tools APIs in 2019. *Nucleic Acids Res.* 47 W636–W641. 10.1093/nar/gkz268 30976793 PMC6602479

[B46] Mardanov A. V. Glukhova L. B. Gruzdev E. V. Beletsky A. V. Karnachuk O. V. Ravin N. V. (2016). The complete mitochondrial genome of the acid-tolerant fungus Penicillium ShG4C. *Genomics Data* 10 141–143. 10.1016/j.gdata.2016.11.009 27872815 PMC5109282

[B47] Mattiazzi M. Petrovic U. Krizaj I. (2012). Yeast as a model eukaryote in toxinology: a functional genomics approach to studying the molecular basis of action of pharmacologically active molecules. *Toxicon* 60 558–571. 10.1016/j.toxicon.2012.03.014 22465496

[B48] Mistry J. Chuguransky S. Williams L. Qureshi M. Salazar G. A. Sonnhammer E. L. L. (2021). Pfam: The protein families database in 2021. *Nucleic Acids Res.* 49 D412–D419. 10.1093/nar/gkaa913 33125078 PMC7779014

[B49] Nakatsu C. H. Barabote R. Thompson S. Bruce D. Detter C. Brettin T. (2013). Complete genome sequence of Arthrobacter sp strain FB24. *Stand. Genomic Sci.* 9 106–116. 10.4056/sigs.4438185 24501649 PMC3910542

[B50] Naveed S. Li C. H. Zhang J. Y. Zhang C. H. Ge Y. (2020). Sorption and transformation of arsenic by extracellular polymeric substances extracted from Synechocystis sp. PCC6803. *Ecotoxicol. Environ. Saf.* 206:111200. 10.1016/j.ecoenv.2020.111200 32889308

[B51] Nayfa M. G. Jones D. B. Benzie J. A. H. Jerry D. R. Zenger K. R. (2020). Comparing Genomic Signatures of Selection Between the Abbassa Strain and Eight Wild Populations of Nile Tilapia (Oreochromis niloticus) in Egypt. *Front. Genet.* 11:567969. 10.3389/fgene.2020.567969 33193660 PMC7593532

[B52] Niegowski D. Eshaghi S. (2007). The CorA family: structure and function revisited. *Cell. Mol. Life Sci. CMLS* 64 2564–2574. 10.1007/s00018-007-7174-z 17619822 PMC11136245

[B53] Nies A. Nies D. H. Silver S. (1990). Nucleotide sequence and expression of a plasmid-encoded chromate resistance determinant from Alcaligenes eutrophus. *J. Biol. Chem.* 265 5648–5653. 2180932

[B54] Patel R. K. Jain M. (2012). NGS QC Toolkit: A Toolkit for Quality Control of Next Generation Sequencing Data. *PLoS One* 7:30619. 10.1371/journal.pone.0030619 22312429 PMC3270013

[B55] Pathak A. Kothari R. Vinoba M. Habibi N. Tyagi V. V. (2021). Fungal bioleaching of metals from refinery spent catalysts: A critical review of current research, challenges, and future directions. *J. Environ. Manage.* 280:111789. 10.1016/j.jenvman.2020.111789 33370668

[B56] Peng L. Li L. W. Liu X. C. Chen J. W. Shi C. C. Guo W. J. (2020). Chromosome-Level Comprehensive Genome of Mangrove Sediment-Derived Fungus Penicillium variabile HXQ-H-1. *J. Fungi* 6:12. 10.3390/jof6010007 31878043 PMC7151134

[B57] Peng M. Dilokpimol A. Makela M. R. Hilden K. Bervoets S. Riley R. (2017). The draft genome sequence of the ascomycete fungus Penicillium subrubescens reveals a highly enriched content of plant biomass related CAZymes compared to related fungi. *J. Biotechnol.* 246 1–3. 10.1016/j.jbiotec.2017.02.012 28216099

[B58] Qi C. L. Wang X. D. Han F. L. Chen X. F. Li E. C. Zhang M. L. (2021). Dietary arginine alleviates the oxidative stress, inflammation and immunosuppression of juvenile Chinese mitten crab Eriocheir sinensis under high pH stress. *Aquacult. Rep.* 19:8. 10.1016/j.aqrep.2021.100619

[B59] Samanta S. Singh A. Banerjee A. Roychoudhury A. (2020). Exogenous supplementation of melatonin alters representative organic acids and enzymes of respiratory cycle as well as sugar metabolism during arsenic stress in two contrasting indica rice cultivars. *J. Biotechnol.* 324 220–232. 10.1016/j.jbiotec.2020.10.013 33068698

[B60] Schafhauser T. Wibberg D. Ruckert C. Winkler A. Flor L. van Pee K. H. (2015). Draft genome sequence of Talaromyces islandicus (”Penicillium islandicum”) WF-38-12, a neglected mold with significant biotechnological potential. *J. Biotechnol.* 211 101–102. 10.1016/j.jbiotec.2015.07.004 26197417

[B61] Schattner P. Brooks A. N. Lowe T. M. (2005). The tRNAscan-SE, snoscan and snoGPS web servers for the detection of tRNAs and snoRNAs. *Nucleic Acids Res.* 33 W686–W689. 10.1093/nar/gki366 15980563 PMC1160127

[B62] Sriharsha D. V. Kumar R. L. Janakiraman S. (2020). Absorption and Reduction of Chromium by Fungi. *Bull. Environ. Contam. Toxicol.* 105 645–649. 10.1007/s00128-020-02979-7 32870333

[B63] Sun J. X. Wang S. C. Cao Y. R. Wang S. T. Li S. (2020). Cadmium exposure induces apoptosis, inflammation and immunosuppression through CYPs activation and antioxidant dysfunction in common carp neutrophils. *Fish Shellf. Immunol.* 99 284–290. 10.1016/j.fsi.2020.02.015 32058096

[B64] Wang Y. P. Tang H. B. DeBarry J. D. Tan X. Li J. P. Wang X. Y. (2012). MCScanX: a toolkit for detection and evolutionary analysis of gene synteny and collinearity. *Nucleic Acids Res.* 40:gkr1293. 10.1093/nar/gkr1293 22217600 PMC3326336

[B65] Wang Y. S. Liang H. L. Chen G. P. Liao C. G. Wang Y. C. Hu Z. L. (2019). Molecular and Phylogenetic Analyses of the Mediator Subunit Genes in Solanum lycopersicum. *Front. Genet.* 10:01222. 10.3389/fgene.2019.01222 31827491 PMC6892441

[B66] Wingenfelder U. Hansen C. Furrer G. Schulin R. (2005). Removal of heavy metals from mine waters by natural zeolites. *Environ. Sci. Technol.* 39 4606–4613. 10.1021/es048482s 16047799

[B67] Xu H. B. Luo X. Qian J. Pang X. H. Song J. Y. Qian G. R. (2012). FastUniq: A Fast De Novo Duplicates Removal Tool for Paired Short Reads. *PLoS One* 7:6. 10.1371/journal.pone.0052249 23284954 PMC3527383

[B68] Xu X. Y. Hao R. X. Xu H. Lu A. H. (2020). Removal mechanism of Pb(II) by Penicillium polonicum: immobilization, adsorption, and bioaccumulation. *Sci. Rep.* 10:9079. 10.1038/s41598-020-66025-6 32493948 PMC7270113

[B69] Yan W. Z. Wang N. Wei D. Liang C. Y. Chen X. M. Liu L. (2021). Bacterial community compositions and nitrogen metabolism function in a cattle farm wastewater treatment plant revealed by Illumina high-throughput sequencing. *Environ. Sci. Pollut. Res.* 13 [preprint]. 10.1007/s11356-021-13570-w 33772473

[B70] Yang Y. Chen M. Li Z. W. Al-Haimi A. M. S. de Hoog S. Pan W. H. (2016). Genome Sequencing and Comparative Genomics Analysis Revealed Pathogenic Potential in Penicillium capsulatum as a Novel Fungal Pathogen Belonging to Eurotiales. *Front. Microbiol.* 7:01541. 10.3389/fmicb.2016.01541 27761131 PMC5051111

[B71] Yin K. Wang Q. N. Lv M. Chen L. X. (2019). Microorganism remediation strategies towards heavy metals. *Chem. Eng. J.* 360 1553–1563. 10.1016/j.cej.2018.10.226

[B72] Zhang L. F. Chen Y. Y. Zhang W. J. (2011). Removal of Cr(VI) from Aqueous Solution by Acid Treated Fungal Biomass. *Adv. Mater. Res.* 197-198 131–135. 10.4028/www.scientific.net/AMR.197-198.131

[B73] Zhang L. L. Huang W. Zhang Y. Y. Fan G. He J. Ren J. N. (2020). Genomic and Transcriptomic Study for Screening Genes Involved in the Limonene Biotransformation of Penicillium digitatum DSM 62840. *Front. Microbiol.* 11:744. 10.3389/fmicb.2020.00744 32390984 PMC7188761

[B74] Zhou J. F. Zeng Z. Q. Gao Y. G. Yu Z. H. (2008). The Summary of the Database of Fungal Genomes Publicly Available. *Instit. Microbiol.* 35 1311–1318. 10.13344/j.microbiol.china.2008.08.008

[B75] Zhou Y. Huang X. T. Huang G. H. Bai X. B. Tang X. L. Li Y. Z. (2008). Cu and Fe Bioleaching in Low-grade Chalcopyrite and Bioleaching Mechanisms Using Penicillium janthinellum Strain GXCR. *Chin. J. Biotechnol.* 24 1993–2002. 19256351

