# Supplementary material for: Sequencing and Comparative Genomic Analysis of a Highly Metal-Tolerant Penicillium janthinellum P1 Provide Insights Into Its Metal Tolerance
Source: Front Microbiol. 2021 Jun 4;12:663217. doi: 10.3389/fmicb.2021.663217 (PMC8212970; doi:10.3389/fmicb.2021.663217)
Supplement: Supplementary file 1 [file Data_Sheet_1.docx]

**Supplementary Figure 1 |** Interspecies collinearity map made by MCScanX.(a) *P. janthinellum* P1 and *P. janthinellum* ATCC 10455; (b) *P. janthinellum* P1 and *P. oxalicum* 114-2

**
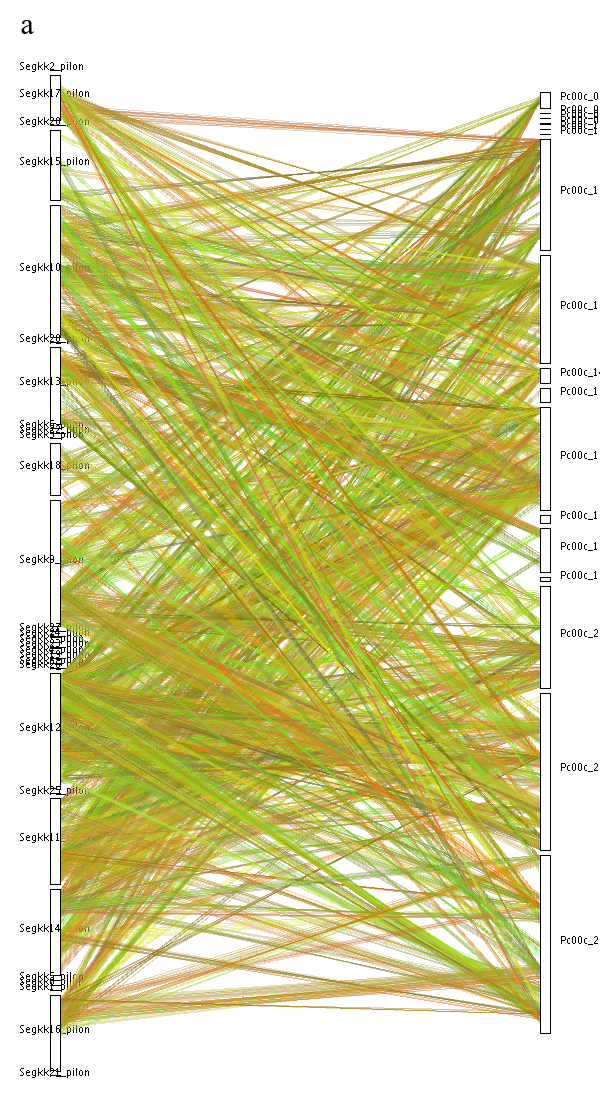

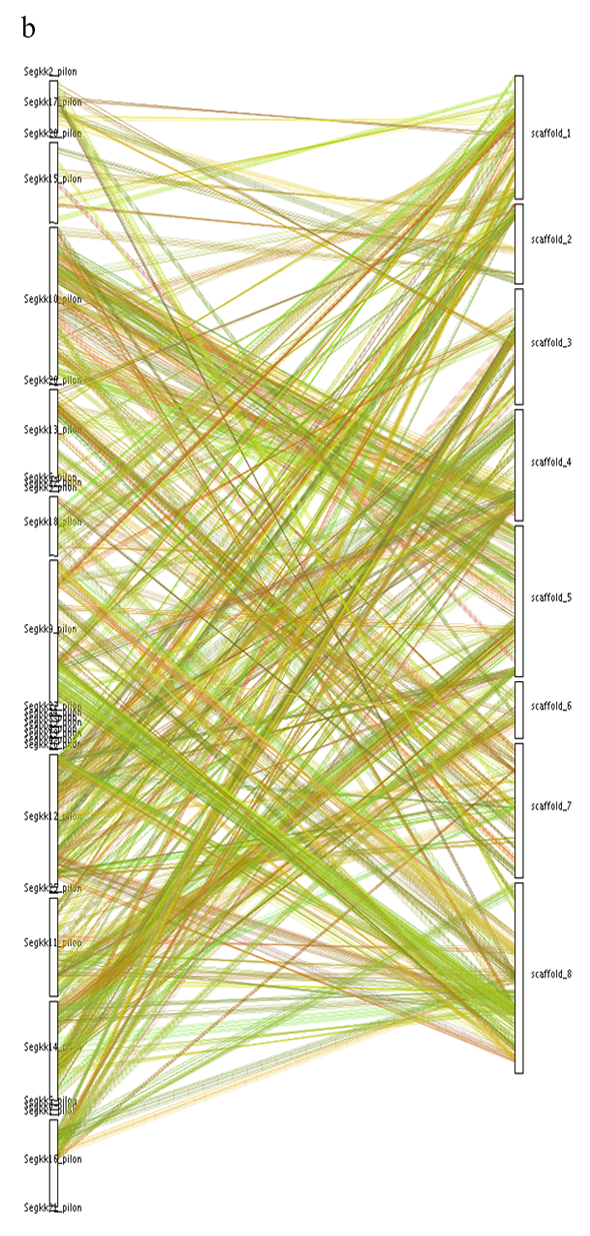
**

**Supplementary Figure 2 |** Gene alignment map using Mauve.


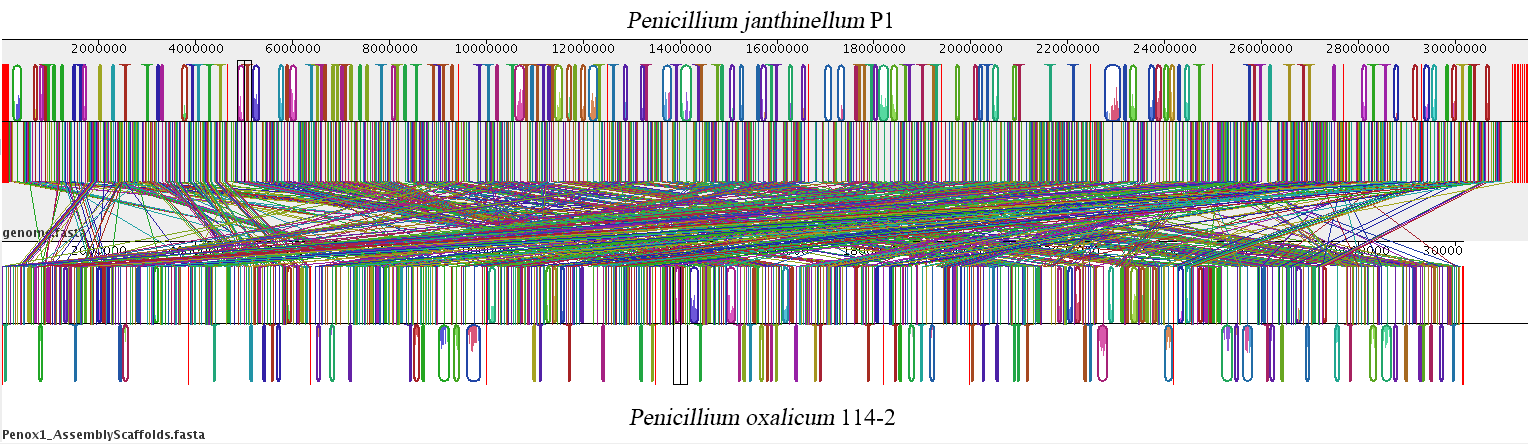


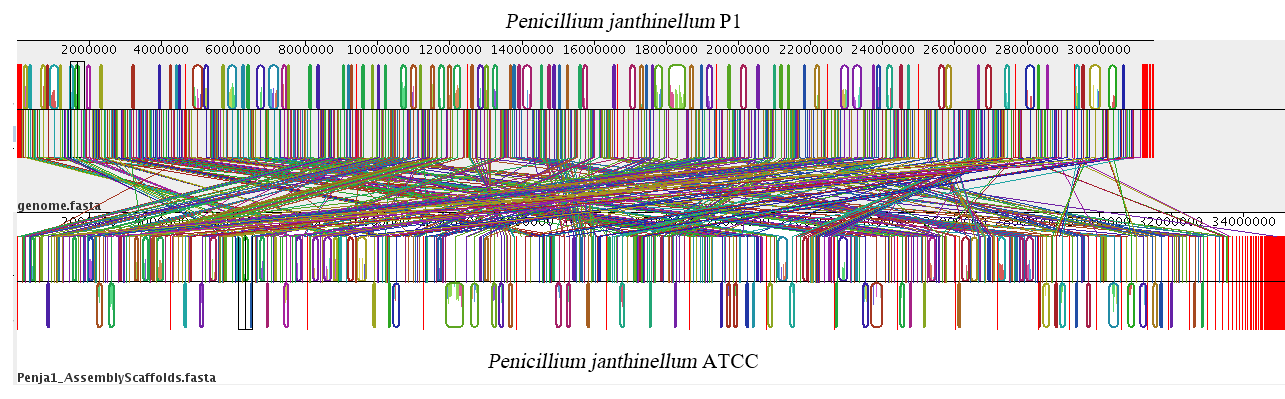


The line above indicates the position of the genome in P1. The rectangles represent the genome fragments and the white gaps between the rectangles mean no similar fragments were found in the other genome.

**Supplementary Table 1 |** The top 20 annotated species in NCBI.

| Magir Annotated species | Number |
| --- | --- |
| Penicillium chrysogenum | 1752 |
| Penicillium arizonense | 1165 |
| Penicillium expansum | 1046 |
| Aspergillus niger | 898 |
| Penicillium digitatum | 796 |
| Aspergillus oryzae | 694 |
| Aspergillus piperis | 598 |
| Aspergillus welwitschiae | 598 |
| Aspergillus lacticoffeatus | 587 |
| Aspergillus costaricaensis | 585 |
| Aspergillus vadensis | 585 |
| Aspergillus neoniger | 580 |
| Aspergillus eucalypticola | 568 |
| Aspergillus aculeatinus | 521 |
| Aspergillus ibericus | 494 |
| Aspergillus terreus | 486 |
| Aspergillus japonicus | 485 |
| Aspergillus brunneoviolaceus | 478 |
| Aspergillus heteromorphus | 471 |
| Aspergillus sclerotioniger | 466 |

**Supplementary Table 2 |** KEGG Metabolic pathway classification of P1.

| KEGG classification | Metabolic pathway | Number | Path number |
| --- | --- | --- | --- |
| Amino acid metabolism | Alanine, aspartate and glutamate metabolism | 11 | ko00250 |
|  | Glycine, serine and threonine metabolism | 9 | ko00260 |
|  | Cysteine and methionine metabolism | 7 | ko00270 |
|  | Valine, leucine and isoleucine degradation | 3 | ko00280 |
|  | Valine, leucine and isoleucine biosynthesis | 4 | ko00290 |
|  | Lysine biosynthesis | 3 | ko00300 |
|  | Lysine degradation | 2 | ko00310 |
|  | Arginine biosynthesis | 7 | ko00220 |
|  | Arginine and proline metabolism | 7 | ko00330 |
|  | Histidine metabolism | 3 | ko00340 |
|  | Tyrosine metabolism | 4 | ko00350 |
|  | Phenylalanine metabolism | 4 | ko00360 |
|  | Tryptophan metabolism | 5 | ko00380 |
|  | Phenylalanine, tyrosine and tryptophan biosynthesis | 5 | ko00400 |
|  | beta-Alanine metabolism | 2 | ko00410 |
|  | Taurine and hypotaurine metabolism | 1 | ko00430 |
|  | Selenocompound metabolism | 5 | ko00450 |
|  | Cyanoamino acid metabolism | 3 | ko00460 |
|  | Glutathione metabolism | 4 | ko00480 |
| Biosynthesis of other secondary metabolites | Caffeine metabolism | 2 | ko00232 |
|  | Aflatoxin biosynthesis | 1 | ko00254 |
| Carbohydrate metabolism | Glycolysis/Gluconeogenesis | 7 | ko00010 |
|  | Citrate cycle (TCA cycle) | 6 | ko00020 |
|  | Pentose phosphate pathway | 4 | ko00030 |
|  | Pentose and glucuronate interconversions | 3 | ko00040 |
|  | Fructose and mannose metabolism | 6 | ko00051 |
|  | Galactose metabolism | 5 | ko00052 |
|  | Ascorbate and aldarate metabolism | 2 | ko00053 |
|  | Starch and sucrose metabolism | 14 | ko00500 |
|  | Amino sugar and nucleotide sugar metabolism | 9 | ko00520 |
|  | Pyruvate metabolism | 11 | ko00620 |
|  | Glyoxylate and dicarboxylate metabolism | 7 | ko00630 |
| Energy Metabolism | Oxidative phosphorylation | 7 | ko00190 |
|  | Nitrogen metabolism | 6 | ko00910 |
|  | Sulfur metabolism | 5 | ko00920 |
|  | Carbon fixation in photosynthetic organisms | 4 | ko00710 |
|  | Carbon fixation pathways in prokaryotes | 4 | ko00720 |
| Sugar chain biosynthesis and metabolism | N-Glycan biosynthesis | 4 | ko00510 |
|  | Various types of N-glycan biosynthesis | 2 | ko00513 |
|  | Other types of O-glycan biosynthesis | 1 | ko00514 |
|  | Mannose type O-glycan biosynthesis | 1 | ko00515 |
|  | Other glycan degradation | 1 | ko00511 |
| Fat metabolisim | Fatty acid biosynthesis | 4 | ko00061 |
|  | Fatty acid degradation | 3 | ko00071 |
|  | Glycerolipid metabolism | 2 | ko00561 |
|  | Primary bile acid biosynthesis | 1 | ko00120 |
|  | Glycerophospholipid metabolism | 5 | ko00564 |
|  | Ether lipid metabolism | 1 | ko00565 |
|  | Arachidonic acid metabolism | 1 | ko00590 |
|  | Sphingolipid metabolism | 2 | ko00600 |
|  | Biosynthesis of unsaturated fatty acids | 2 | ko01040 |
| Cofactor and vitamin metabolism | Thiamine metabolism | 2 | ko00730 |
|  | Nicotinate and nicotinamide metabolism | 3 | ko00760 |
|  | Pantothenate and CoA biosynthesis | 1 | ko00770 |
|  | Folate biosynthesis | 1 | ko00790 |
|  | One carbon pool by folate | 4 | ko00670 |
|  | Porphyrin and chlorophyll metabolism | 2 | ko00860 |
|  | Ubiquinone and other terpenoid-quinone biosynthesis | 1 | ko00130 |
| Metabolism of terpenoids and polyketides | Sesquiterpenoid and triterpenoid biosynthesis | 1 | ko00909 |
|  | Terpenoid backbone biosynthesis | 3 | ko00900 |
| Nucleotide metabolism | Purine metabolism | 12 | ko00230 |
|  | Pyrimidine metabolism | 5 | ko00240 |

**Supplementary Table 3 |** Repeated sequence annotation of P1.

| Transposable Elements | Length (bp) | Percentage (%) |
| --- | --- | --- |
| Retroelements | 92935 | 0.29 % |
| SINEs | 1570 | 0.00 % |
| LINEs | 35525 | 0.11 % |
| LTR elements | 55840 | 0.18 % |
| Ty1/Copia | 7810 | 0.02 % |
| Gypsy/DIRS1 | 48030 | 0.15 % |
| DNA transposons | 122416 | 0.39 % |
| hobo-Activator | 8287 | 0.03 % |
| Tc1-IS630-Pogo | 112816 | 0.36 % |
| Tourist/Harbinger | 497 | 0.00 % |
| Unclassified | 884 | 0.00 % |
| Other repeats | 340554 | 1.08 % |
| Satellites | 273 | 0.00 % |
| Simple repeats | 275606 | 0.87 % |
| Low complexity | 64675 | 0.21 % |
| Total | 599497 | 1.90 % |

**Supplementary Table 4 |** Occurrence of simple sequence repeats (SRRs) of P1.

| SSR type | Number | Percent (%) | Total length (bp) | Averge length (bp) |
| --- | --- | --- | --- | --- |
| 1 | 2919 | 53.5 | 38463 | 13 |
| 2 | 739 | 13.5 | 13500 | 18 |
| 3 | 476 | 8.7 | 10248 | 22 |
| 4 | 47 | 0.9 | 1224 | 26 |
| 5 | 24 | 0.4 | 850 | 35 |
| 6 | 21 | 0.4 | 738 | 35 |
| 7 | 1216 | 22.3 | 21123 | 17 |
| 8 | 15 | 0.3 | 692 | 46 |
| Total | 5457 | 100 | 86838 | 16 |

**Supplementary Table 5 |** Type and number of annotated tRNA.

| Ala:11 | AGC:6 | CGC:2 | TGC:3 |  |  |
| --- | --- | --- | --- | --- | --- |
| Gly:13 | GCC:9 | CCC:1 | TCC:3 |  |  |
| Pro:8 | AGG:5 | CGG:1 | TGG:2 |  |  |
| Thr:9 | AGT:6 | CGT:1 | TGT:2 |  |  |
| Val:27 | AAC:7 | CAC:2 | TAC:18 |  |  |
| Ser:9 | AGA:6 | CGA:1 | TGA:2 |  |  |
| Arg:26 | ACG:8 | CCG:2 | TCG:2 | CCT:1 | TCT:13 |
| Leu:10 | AAG:4 | CAG:3 | TAG:2 | TAA:1 |  |
| Phe:22 | GAA:22 |  |  |  |  |
| Asn:16 | GTT:16 |  |  |  |  |
| Lys:12 | CTT:7 | TTT:5 |  |  |  |
| Asp:8 | GTC:8 |  |  |  |  |
| Glu:25 | CTC:7 | TTC:18 |  |  |  |
| His:21 | GTG:21 |  |  |  |  |
| Gln:7 | CTG:5 | TTG:2 |  |  |  |
| Ile:24 | AAT:8 | GAT:14 | TAT:2 |  |  |
| Met:3 | CAT:3 |  |  |  |  |
| Tyr:4 | GTA:4 |  |  |  |  |
| Cys:3 | GCA:3 |  |  |  |  |
| Trp:2 | CCA:2 |  |  |  |  |
| SelCys:14 | TCA:14 |  |  |  |  |

**Supplementary Table 6 |** Type and number of annotated rRNA.

| NcRNA type | Loci | Average length (bp) | Total length (bp) | Percent of genome (%) |
| --- | --- | --- | --- | --- |
| tRNA | 503 | 65 | 32595 | 0.10 % |
| rRNA (5S) | 35 | 115 | 4024 | 0.01 % |
| rRNA (5.8S) | 8 | 143 | 1146 | 0.00 % |
| rRNA (18S) | 7 | 1792 | 12545 | 0.04 % |
| rRNA (28S) | 20 | 2706 | 54124 | 0.17 % |
| snoRNA | 14 | 99 | 1390 | 0.00 % |

**Supplementary Table 7 |** Resources of the other 24 fungi for OrthoMCL analysis.

| Abbreviation | Species | Downloading website |
| --- | --- | --- |
| pench | *Penicillium chrysogenum* Wisconsin 54-1255 | <https://mycocosm.jgi.doe.gov/PenchWisc1_1/PenchWisc1_1.home.html> |
| penex | *Penicillium expansum* ATCC 24692 | <https://mycocosm.jgi.doe.gov/Penex1/Penex1.home.html> |
| aspni | *Aspergillus niger* NRRL3 | <https://mycocosm.jgi.doe.gov/Aspni_NRRL3_1/Aspni_NRRL3_1.home.html> |
| pendi | *Penicillium digitatum* PHI26 | <https://mycocosm.jgi.doe.gov/Pendi1/Pendi1.home.html> |
| aspor | *Aspergillus oryzae* RIB40 | <https://mycocosm.jgi.doe.gov/Aspor1/Aspor1.home.html> |
| asppi | *Aspergillus piperis* CBS 112811 | <https://mycocosm.jgi.doe.gov/Asppip1/Asppip1.home.html> |
| aspwe | *Aspergillus welwitschiae* CBS 101883 | <https://mycocosm.jgi.doe.gov/Asplac1/Asplac1.home.html> |
| aspla | *Aspergillus lacticoffeatus* CBS139.54b | <https://mycocosm.jgi.doe.gov/Aspwel1/Aspwel1.home.html> |
| aspco | *Aspergillus costaricaensis* CBS 115574 | <https://mycocosm.jgi.doe.gov/Aspcos1/Aspcos1.home.html> |
| aspva | *Aspergillus vadensis* CBS 113365 | <https://mycocosm.jgi.doe.gov/Aspvad1/Aspvad1.home.html> |
| aspne | *Aspergillus neoniger* CBS 115656 | <https://mycocosm.jgi.doe.gov/Aspneo1/Aspneo1.home.html> |
| aspeu | *Aspergillus eucalypticola* CBS 122712 | <https://mycocosm.jgi.doe.gov/Aspeuc1/Aspeuc1.home.html> |
| aspac | *Aspergillus aculeatinus* CBS 121060 | <https://mycocosm.jgi.doe.gov/Aspacu1/Aspacu1.home.html> |
| aspib | *Aspergillus ibericus* CBS 121593 | <https://mycocosm.jgi.doe.gov/Aspibe1/Aspibe1.home.html> |
| aspte | *Aspergillus terreus* NIH 2624 | <https://mycocosm.jgi.doe.gov/Aspte1/Aspte1.home.html> |
| aspja | *Aspergillus japonicus* CBS 114.51 | <https://mycocosm.jgi.doe.gov/Aspjap1/Aspjap1.home.html> |
| aspbr | *Aspergillus brunneoviolaceus* CBS 621.78 | <https://mycocosm.jgi.doe.gov/Aspbru1/Aspbru1.home.html> |
| asphe | *Aspergillus heteromorphus* CBS 117.55 | <https://mycocosm.jgi.doe.gov/Asphet1/Asphet1.home.html> |
| aspsc | *Aspergillus sclerotioniger* CBS115572 | <https://mycocosm.jgi.doe.gov/Aspscl1/Aspscl1.home.html> |
| aspcl | *Aspergillus clavatus* NRRL 1 | <https://mycocosm.jgi.doe.gov/Aspcl1/Aspcl1.home.html> |
| sacce | *Saccharomyces cerevisiae* YB210 | <https://mycocosm.jgi.doe.gov/SacceYB210_1/SacceYB210_1.home.html> |
| tripa | *Trichoderma parareesei* CBS 125925 | <https://mycocosm.jgi.doe.gov/Tripar1/Tripar1.home.html> |
| penox | *Penicillium oxalicum* 114-2 | <https://mycocosm.jgi.doe.gov/Penox1/Penox1.home.html> |
| tubme | *Tuber melanosporum* Mel28 | <https://mycocosm.jgi.doe.gov/Tubme1v2/Tubme1v2.home.html> |

**Supplementary Table 8 |** Orthologous gene number of 25 fungi.

|  | 25 | 24 | 23 | 22 | 21 | 20 | 19 | 18 | 17 | 16 | 15 | 14 | 13 | 12 | 11 | 10 | 9 | 8 | 7 | 6 | 5 | 4 | 3 | 2 | 1 |
| --- | --- | --- | --- | --- | --- | --- | --- | --- | --- | --- | --- | --- | --- | --- | --- | --- | --- | --- | --- | --- | --- | --- | --- | --- | --- |
| aspco | 2142 | 1761 | 1124 | 870 | 499 | 386 | 332 | 333 | 357 | 357 | 337 | 333 | 252 | 307 | 290 | 251 | 218 | 268 | 158 | 130 | 162 | 186 | 191 | 240 | 427 |
| aspne | 2142 | 1746 | 1122 | 871 | 496 | 379 | 331 | 323 | 353 | 352 | 328 | 326 | 255 | 305 | 275 | 231 | 196 | 253 | 147 | 126 | 139 | 186 | 187 | 260 | 388 |
| aspeu | 2138 | 1745 | 1117 | 876 | 489 | 368 | 327 | 318 | 351 | 341 | 318 | 310 | 242 | 293 | 285 | 238 | 180 | 243 | 128 | 109 | 124 | 160 | 167 | 269 | 506 |
| aspva | 2138 | 1754 | 1117 | 873 | 494 | 382 | 330 | 334 | 350 | 346 | 335 | 319 | 252 | 302 | 289 | 245 | 189 | 264 | 138 | 156 | 153 | 170 | 176 | 268 | 545 |
| aspla | 2140 | 1761 | 1135 | 868 | 503 | 384 | 341 | 326 | 348 | 349 | 341 | 328 | 256 | 320 | 299 | 234 | 220 | 267 | 160 | 139 | 165 | 215 | 330 | 536 | 740 |
| aspni | 2233 | 1785 | 1157 | 890 | 519 | 395 | 351 | 351 | 373 | 367 | 354 | 341 | 264 | 328 | 328 | 254 | 218 | 270 | 139 | 115 | 128 | 134 | 167 | 155 | 7 |
| aspwe | 2135 | 1757 | 1132 | 871 | 489 | 378 | 332 | 331 | 347 | 356 | 331 | 343 | 262 | 315 | 297 | 247 | 215 | 273 | 142 | 130 | 170 | 200 | 380 | 585 | 1061 |
| aspib | 2144 | 1745 | 1102 | 858 | 496 | 376 | 323 | 301 | 328 | 320 | 277 | 276 | 209 | 239 | 244 | 199 | 134 | 100 | 95 | 119 | 117 | 129 | 133 | 236 | 831 |
| aspsc | 2152 | 1753 | 1128 | 874 | 491 | 383 | 328 | 313 | 340 | 345 | 309 | 301 | 232 | 255 | 256 | 213 | 136 | 129 | 120 | 126 | 154 | 159 | 192 | 288 | 1200 |
| asphe | 2111 | 1720 | 1082 | 842 | 445 | 322 | 254 | 236 | 250 | 239 | 193 | 188 | 129 | 161 | 166 | 88 | 77 | 63 | 75 | 81 | 79 | 124 | 162 | 235 | 1304 |
| aspbr | 2142 | 1754 | 1114 | 858 | 490 | 378 | 311 | 316 | 333 | 295 | 251 | 251 | 173 | 153 | 144 | 92 | 129 | 108 | 128 | 162 | 188 | 210 | 665 | 381 | 745 |
| aspac | 2155 | 1753 | 1120 | 876 | 496 | 362 | 318 | 311 | 325 | 296 | 260 | 256 | 179 | 151 | 139 | 97 | 126 | 118 | 142 | 159 | 187 | 218 | 657 | 395 | 632 |
| aspja | 2157 | 1757 | 1116 | 873 | 496 | 378 | 303 | 305 | 329 | 308 | 263 | 254 | 166 | 151 | 137 | 101 | 122 | 120 | 141 | 172 | 181 | 209 | 661 | 237 | 705 |
| aspte | 2199 | 1728 | 1032 | 783 | 389 | 275 | 242 | 212 | 242 | 189 | 151 | 129 | 106 | 147 | 120 | 94 | 104 | 118 | 141 | 141 | 133 | 158 | 192 | 240 | 34 |
| aspor | 2260 | 1731 | 1030 | 806 | 389 | 309 | 272 | 272 | 297 | 256 | 233 | 176 | 111 | 183 | 153 | 133 | 150 | 148 | 151 | 190 | 186 | 226 | 209 | 291 | 116 |
| aspcl | 2188 | 1737 | 1069 | 810 | 401 | 266 | 170 | 179 | 182 | 136 | 119 | 78 | 79 | 81 | 83 | 72 | 81 | 76 | 103 | 92 | 88 | 106 | 76 | 142 | 10 |
| penex | 2204 | 1768 | 1142 | 887 | 497 | 355 | 308 | 258 | 189 | 181 | 148 | 135 | 106 | 156 | 125 | 119 | 161 | 130 | 205 | 201 | 277 | 290 | 479 | 410 | 658 |
| pendi | 2147 | 1702 | 1033 | 785 | 371 | 232 | 151 | 134 | 108 | 79 | 65 | 46 | 43 | 60 | 72 | 66 | 76 | 71 | 97 | 140 | 162 | 166 | 345 | 202 | 42 |
| pench | 2233 | 1780 | 1102 | 875 | 474 | 354 | 279 | 230 | 199 | 158 | 137 | 120 | 122 | 130 | 114 | 110 | 161 | 176 | 177 | 212 | 285 | 311 | 519 | 560 | 2316 |
| **penja** | 2217 | 1776 | 1122 | 867 | 466 | 340 | 281 | 226 | 191 | 156 | 145 | 132 | 104 | 130 | 109 | 92 | 105 | 112 | 142 | 159 | 238 | 218 | 220 | 210 | 18 |
| penox | 2160 | 1733 | 1047 | 811 | 396 | 242 | 166 | 160 | 126 | 102 | 71 | 61 | 57 | 82 | 75 | 52 | 77 | 73 | 101 | 114 | 142 | 121 | 109 | 144 | 23 |
| tripa | 2128 | 1659 | 842 | 353 | 200 | 154 | 119 | 110 | 113 | 103 | 79 | 61 | 56 | 81 | 68 | 55 | 67 | 57 | 74 | 82 | 90 | 113 | 106 | 202 | 134 |
| tubme | 2140 | 1639 | 565 | 206 | 116 | 258 | 164 | 72 | 51 | 66 | 66 | 20 | 62 | 35 | 56 | 34 | 21 | 37 | 42 | 70 | 88 | 108 | 59 | 192 | 1080 |
| sacce | 2269 | 534 | 143 | 69 | 44 | 32 | 27 | 31 | 20 | 15 | 13 | 9 | 3 | 12 | 13 | 9 | 7 | 4 | 5 | 8 | 6 | 3 | 20 | 42 | 2236 |

Column 25: number of genes which have orthologous genes in 25 species; column 24: number of genes which have orthologous genes in 24 species and so on. The smaller the number of column 1 of a certain species, the higher the degree of similarity with these 25 species. *Saccharomyces cerevisiae*, as an outgroup species, has a particularly large number of columns 1 in the total number of genes. In contrast, penja has a small proportion, indicating that it is very similar to the selected.

**Supplementary Table 9 |** List of the CDS with the predicted function as the compositions of the secretion system.

The proteins encoded by P1 genome that were predicted as the compositions of the eukaryotic Sec-SRP secretion systems were list and the function of the corresponding gene functions were indicated.

| **CDS** | **Putative gene function** |
| --- | --- |
| penja_456 | Signal recognition particle subunit SRP14 |
| penja_1983 | Signal recognition particle receptor subunit beta |
| penja_4564 | Signal recognition particle subunit SRP72 |
| penja_5876 | Signal recognition particle Sec65 subunit |
| penja_10324 | Signal recognition particle subunit SRP68 |
| penja_88 | Protein transport protein Sec61 subunit beta |
| penja_126 | Protein transport protein Sec23 |
| penja_172 | protein transport protein Sec61 subunit gamma |
| penja_387 | Protein transport protein Sec24 |
| penja_1366 | Protein transport protein Sec39 |
| penja_1654 | Protein transport protein Sec24 |
| penja_4236 | Protein transport protein Sec31 |
| penja_5108 | Protein transport protein Sec9 |
| penja_5411 | Protein transport protein Sec7 |
| penja_5663 | Protein transport protein Sec61 |
| penja_6764 | Protein transport protein Sec22 |
| penja_8008 | Protein transport protein Sec1 |
| penja_8362 | Protein transport protein Sec13 |
| penja_10349 | Protein transport protein Sec13 |
| penja_4579 | Translocation protein Sec63 |
| penja_7331 | Translocation protein Sec66 |
| penja_7884 | Translocation protein Sec62 |

**Supplementary Table 10 |** Carboydrate-Active Enzymes of *P. janthinellum* P1, *P. janthinellum* ATCC 10455 and *P. oxalicum* 114-2.

| CAZy_family | *P. janthinellum* P1 | *P. janthinellum* ATCC 10455 | *P. oxalicum* 114-2 |
| --- | --- | --- | --- |
| AA1 | 10 | 9 | 6 |
| AA2 | 3 | 3 | 3 |
| AA3 | 27 | 24 | 12 |
| AA4 | 6 | 4 | 3 |
| AA5 | 2 | 0 | 0 |
| AA6 | 1 | 1 | 1 |
| AA7 | 33 | 32 | 19 |
| AA8 | 2 | 2 | 1 |
| AA9 | 5 | 5 | 4 |
| AA11 | 2 | 3 | 1 |
| AA13 | 1 | 0 | 1 |
| CBM1 | 7 | 26 | 20 |
| CBM13 | 1 | 1 | 0 |
| CBM18 | 5 | 10 | 10 |
| CBM20 | 3 | 4 | 4 |
| CBM21 | 1 | 1 | 1 |
| CBM22 | 0 | 0 | 0 |
| CBM24 | 2 | 6 | 4 |
| CBM32 | 3 | 2 | 0 |
| CBM35 | 1 | 1 | 2 |
| CBM42 | 1 | 1 | 1 |
| CBM43 | 3 | 3 | 2 |
| CBM46 | 1 | 1 | 1 |
| CBM48 | 2 | 2 | 2 |
| CBM50 | 6 | 9 | 11 |
| CBM63 | 1 | 2 | 2 |
| CBM66 | 1 | 0 | 0 |
| CBM67 | 6 | 7 | 2 |
| CE1 | 2 | 4 | 5 |
| CE2 | 0 | 2 | 1 |
| CE3 | 3 | 3 | 0 |
| CE4 | 8 | 7 | 6 |
| CE5 | 7 | 7 | 4 |
| CE8 | 2 | 4 | 4 |
| CE9 | 1 | 2 | 1 |
| CE10 | 0 | 0 | 0 |
| CE12 | 2 | 2 | 2 |
| CE14 | 0 | 2 | 0 |
| CE16 | 2 | 6 | 3 |
| GH1 | 3 | 5 | 4 |
| GH2 | 8 | 9 | 6 |
| GH3 | 21 | 19 | 14 |
| GH5 | 9 | 15 | 9 |
| GH6 | 1 | 2 | 1 |
| GH7 | 3 | 4 | 3 |
| GH10 | 3 | 3 | 3 |
| GH11 | 1 | 7 | 5 |
| GH12 | 3 | 5 | 3 |
| GH13 | 6 | 9 | 4 |
| GH15 | 3 | 5 | 3 |
| GH16 | 15 | 14 | 12 |
| GH17 | 5 | 5 | 5 |
| GH18 | 12 | 19 | 15 |
| GH20 | 2 | 2 | 1 |
| GH23 | 1 | 1 | 1 |
| GH24 | 1 | 1 | 0 |
| GH25 | 1 | 2 | 1 |
| GH26 | 0 | 1 | 1 |
| GH27 | 3 | 6 | 2 |
| GH28 | 15 | 20 | 11 |
| GH29 | 2 | 2 | 0 |
| GH30 | 1 | 5 | 4 |
| GH31 | 9 | 7 | 6 |
| GH32 | 3 | 10 | 2 |
| GH33 | 1 | 2 | 0 |
| GH35 | 5 | 8 | 3 |
| GH36 | 1 | 3 | 0 |
| GH37 | 1 | 1 | 1 |
| GH38 | 1 | 2 | 1 |
| GH42 | 1 | 0 | 0 |
| GH43 | 8 | 11 | 8 |
| GH45 | 0 | 1 | 1 |
| GH47 | 6 | 5 | 6 |
| GH51 | 3 | 5 | 3 |
| GH53 | 1 | 1 | 1 |
| GH54 | 3 | 2 | 1 |
| GH55 | 5 | 6 | 3 |
| GH62 | 2 | 2 | 2 |
| GH63 | 1 | 1 | 1 |
| GH64 | 1 | 1 | 1 |
| GH65 | 1 | 1 | 1 |
| GH67 | 1 | 1 | 1 |
| GH71 | 3 | 8 | 5 |
| GH72 | 6 | 6 | 5 |
| GH75 | 2 | 3 | 3 |
| GH76 | 10 | 8 | 8 |
| GH78 | 10 | 13 | 4 |
| GH79 | 3 | 7 | 2 |
| GH81 | 1 | 1 | 1 |
| GH84 | 1 | 1 | 1 |
| GH88 | 2 | 3 | 1 |
| GH89 | 1 | 1 | 1 |
| GH92 | 6 | 5 | 4 |
| GH93 | 4 | 3 | 3 |
| GH95 | 1 | 3 | 1 |
| GH105 | 4 | 2 | 1 |
| GH106 | 1 | 1 | 0 |
| GH109 | 8 | 3 | 5 |
| GH114 | 2 | 2 | 2 |
| GH115 | 1 | 1 | 0 |
| GH125 | 1 | 2 | 1 |
| GH127 | 1 | 2 | 1 |
| GH128 | 2 | 2 | 1 |
| GH131 | 0 | 1 | 1 |
| GH132 | 2 | 2 | 2 |
| GH133 | 1 | 1 | 1 |
| GH134 | 1 | 1 | 0 |
| GH135 | 1 | 1 | 2 |
| GH140 | 1 | 0 | 0 |
| GH142 | 1 | 0 | 0 |
| GT1 | 8 | 6 | 5 |
| GT2 | 17 | 15 | 15 |
| GT3 | 1 | 1 | 1 |
| GT4 | 7 | 8 | 7 |
| GT5 | 3 | 3 | 2 |
| GT8 | 5 | 5 | 4 |
| GT15 | 3 | 3 | 3 |
| GT20 | 5 | 6 | 5 |
| GT21 | 1 | 1 | 1 |
| GT22 | 4 | 4 | 4 |
| GT24 | 1 | 1 | 1 |
| GT25 | 1 | 1 | 1 |
| GT28 | 0 | 0 | 0 |
| GT31 | 6 | 6 | 3 |
| GT32 | 7 | 9 | 8 |
| GT33 | 1 | 1 | 1 |
| GT34 | 2 | 4 | 3 |
| GT35 | 1 | 1 | 1 |
| GT39 | 3 | 3 | 3 |
| GT41 | 1 | 2 | 2 |
| GT48 | 1 | 1 | 1 |
| GT50 | 1 | 1 | 1 |
| GT54 | 0 | 0 | 0 |
| GT57 | 2 | 2 | 2 |
| GT58 | 1 | 1 | 1 |
| GT59 | 1 | 1 | 1 |
| GT62 | 3 | 3 | 3 |
| GT66 | 1 | 1 | 1 |
| GT69 | 3 | 4 | 4 |
| GT71 | 3 | 4 | 4 |
| GT76 | 1 | 1 | 1 |
| GT90 | 10 | 6 | 7 |
| PL1 | 4 | 4 | 3 |
| PL3 | 1 | 0 | 0 |
| PL4 | 2 | 3 | 3 |
| PL20 | 1 | 0 | 0 |
| PL22 | 0 | 0 | 0 |
| PL26 | 0 | 1 | 1 |
| AAs | 92 | 83 | 51 |
| CBMs | 44 | 76 | 62 |
| CEs | 27 | 39 | 26 |
| GHs | 250 | 314 | 206 |
| GTs | 104 | 105 | 96 |
| PLs | 8 | 8 | 7 |
| Total number | 525 | 625 | 448 |

**Supplementary Table 11 |** Comparison of sugar metabolism genes among *P. janthinellum* P1, *P. janthinellum* ATCC 10455 and *P. oxalicum* 114-2.

| Enzyme | EC Number | CAZy Family | *P. janthinellum* P1 | *P. janthinellum* ATCC 10455 | *P. oxalicum* 114-2 |
| --- | --- | --- | --- | --- | --- |
| 1.polysaccharide degrading enzymes | | | | | |
| α-amylase | 3.2.1.1 | GH13 | 6 | 9 | 4 |
| glucan 1,4-α-glucosidase | 3.2.1.3 | GH15 | 3 | 5 | 3 |
| α-glucosidase | 3.2.1.20 | GH13 | 6 | 9 | 4 |
|  |  | GH31 | 9 | 7 | 6 |
|  |  | GH63 | 1 | 1 | 1 |
|  |  | GH76 | 10 | 8 | 8 |
| amylose isomerase | 2.4.1.18 | GH13 | 6 | 9 | 4 |
| lytic starch monooxygenase | 1.14.99.55 | AA13 | 1 | 0 | 1 |
| dextranase | 3.2.1.11 | CBM35 | 1 | 1 | 2 |
|  |  | GH31 | 9 | 7 | 6 |
| chitinase | 3.2.1.14 | GH18 | 12 | 19 | 15 |
| β-glucosidase | 3.2.1.21 | GH1 | 3 | 5 | 4 |
| amylo-α-1,6-glucosidase | 3.2.1.33 | GH13 | 6 | 9 | 4 |
|  |  | GH133 | 1 | 1 | 1 |
| oligoxyloglucan hydrolase | 3.2.1.120 | GH3 | 21 | 19 | 14 |
| 2. glucose degrading enzymes | | | | | |
| glucose oxidase | 1.1.3.4 | AA3 | 27 | 24 | 12 |
| glucooligosaccharide oxidase | 1.1.3.- | AA5 | 2 | 0 | 0 |
|  |  | AA7 | 33 | 32 | 9 |
| glycogen phosphorylase | 2.4.1.1 | GT35 | 1 | 1 | 1 |
| glucuronosyltransferase | 2.4.1.17 | GT1 | 8 | 6 | 5 |
|  |  | GT2 | 17 | 15 | 15 |
| β-glucuronidase | 3.2.1.31 | GH2 | 8 | 9 | 6 |
|  |  | GH30 | 1 | 5 | 4 |
|  |  | GH79 | 3 | 7 | 2 |
| 3. Polysaccharide synthase | | | | | |
| glycogen synthase | 2.4.1.11 | GT3 | 1 | 1 | 1 |
|  |  | GT5 | 3 | 3 | 2 |
| starch synthase | 2.4.1.21 | GT4 | 7 | 8 | 7 |
| 1,3-β-glucan synthase | 2.4.1.34 | GT48 | 1 | 1 | 1 |

**Supplementary Table 12 |** The predicted secondary metabolism gene clusters among *P. janthinellum* P1, *P. janthinellum* ATCC 10455 and *P. oxalicum* 114-2.

| Cluster | *P. janthinellum* P1 | *P. janthinellum* ATCC 10455 | *P. oxalicum* 114-2 |
| --- | --- | --- | --- |
| T1pks | 7 | 8 | 4 |
| T1pks-Nrps | 3 | 3 | 4 |
| T3pks | 0 | 1 | 1 |
| Indole | 0 | 2 | 2 |
| Indole-T1pks | 0 | 1 | 0 |
| Indole-Nrps | 1 | 0 | 4 |
| Nrps | 7 | 7 | 9 |
| Terpene | 2 | 3 | 4 |
| Saccharide | 1 | 1 | 3 |
| Fatty acid | 2 | 2 | 2 |
| Fatty acid-Nrps | 0 | 0 | 2 |
| Lantipeptide | 1 | 0 | 0 |
| Other | 9 | 6 | 7 |
| Total | 33 | 34 | 42 |
